# APOBEC3 mutagenesis drives therapy resistance in breast cancer

**DOI:** 10.1038/s41588-025-02187-1

**Published:** 2025-05-16

**Authors:** Avantika Gupta, Andrea Gazzo, Pier Selenica, Anton Safonov, Fresia Pareja, Edaise M. da Silva, David N. Brown, Hong Shao, Yingjie Zhu, Juber Patel, Juan Blanco-Heredia, Bojana Stefanovska, Michael A. Carpenter, Yanjun Chen, Isabella Vegas, Xin Pei, Denise Frosina, Achim A. Jungbluth, Marc Ladanyi, Giuseppe Curigliano, Britta Weigelt, Nadeem Riaz, Simon N. Powell, Pedram Razavi, Reuben S. Harris, Jorge S. Reis-Filho, Antonio Marra, Sarat Chandarlapaty

**Affiliations:** 1 Human Oncology and Pathogenesis Program, Memorial Sloan Kettering Cancer Center, New York, NY USA; 2 Department of Pathology and Laboratory Medicine, Memorial Sloan Kettering Cancer Center, New York, NY USA; 3 Department of Medicine, Memorial Sloan Kettering Cancer Center, New York, NY USA; 4 Department of Biochemistry and Structural Biology, University of Texas Health San Antonio, San Antonio, TX USA; 5 Howard Hughes Medical Institute, University of Texas Health San Antonio, San Antonio, TX USA; 6 Department of Radiation Oncology, Memorial Sloan Kettering Cancer Center, New York, NY USA; 7 Department of Oncology and Haemato-Oncology, University of Milano, Milan, Italy; 8 Early Drug Development for Innovative Therapies, European Institute of Oncology IRCSS, Milan, Italy; 9 Weill-Cornell Medical College, New York, NY USA

**Keywords:** Breast cancer, Translational research

## Abstract

Acquired genetic alterations drive resistance to endocrine and targeted therapies in metastatic breast cancer; however, the underlying processes engendering these alterations are largely uncharacterized. To identify the underlying mutational processes, we utilized a clinically annotated cohort of 3,880 patient samples with tumor-normal sequencing. Mutational signatures associated with apolipoprotein B mRNA-editing enzyme catalytic polypeptide-like 3 (APOBEC3) enzymes were prevalent and enriched in post-treatment hormone receptor-positive cancers. These signatures correlated with shorter progression-free survival on antiestrogen plus CDK4/6 inhibitor therapy in hormone receptor-positive metastatic breast cancer. Whole-genome sequencing of breast cancer models and paired primary-metastatic samples demonstrated that active APOBEC3 mutagenesis promoted therapy resistance through characteristic alterations such as *RB1* loss. Evidence of APOBEC3 activity in pretreatment samples illustrated its pervasive role in breast cancer evolution. These studies reveal APOBEC3 mutagenesis to be a frequent mediator of therapy resistance in breast cancer and highlight its potential as a biomarker and target for overcoming resistance.

## Abstract (web summary)

Analysis of a large cohort of metastatic breast cancer samples shows that APOBEC mutational signatures are enriched in post-treatment samples. APOBEC activity was also associated with mutations known to drive drug resistance.

## Main

Both endocrine and targeted therapies are used broadly to treat breast cancer; however, their potential for long-term disease control is limited by tumor evolution and acquired genetic alterations that promote drug resistance^1^. In estrogen receptor (ER)-positive cancers, alterations in several genes including ER (*ESR1*)^2,3^, neurofibromin (*NF1*)^4,5^ and v-erb-b2 avian erythroblastic leukemia viral oncogene homolog 2 (*ERBB2*, or HER2)^6^ are common as tumors develop resistance to antiestrogen therapy. Such genetic alterations also occur after resistance to targeted therapies including cyclin dependent kinase 4/6 inhibitors (CDK4/6i), human epidermal growth factor receptor 2 (HER2) inhibitors and phosphoinositide 3-kinase (PI3K) inhibitors^7–12^. The widespread prevalence of these heterogeneous and acquired genetic alterations has proven challenging to overcome despite the development of second-generation inhibitors such as selective ER degraders (SERDs), underscoring the need to understand the underlying processes of genomic instability and tumor evolution.

Detailed analyses of cancer genomes have identified mutational signatures that can promote tumor evolution, and potentially predict treatment strategies^13–15^. In breast cancer, single base substitution (SBS) signatures associated with the activity of APOBEC3 enzymes (COSMIC SBS2 and SBS13) are prevalent, suggesting their relevance to the underlying genomic instability. APOBEC3 proteins are cytidine deaminases that play roles in innate immunity by restricting viral replication and inhibiting retrotransposition^16^. Although APOBEC3 enzymes serve as protective factors against viral infections, their activity has been implicated in breast tumorigenesis and treatment resistance. Previous studies have linked high expression levels of specific APOBEC3 enzymes to inferior outcomes and tamoxifen resistance in ER-positive tumors^17–19^. Moreover, APOBEC3 mutational signatures are enriched in metastatic breast cancer (MBC) compared with primary breast cancers^20,21^, with APOBEC3 mutagenesis proposed to contribute toward clonal evolution during treatment with endocrine therapy (ET)^22^. These studies reveal a potential association of APOBEC3 mutagenesis with tumor progression and raise the possibility that these processes may be ongoing and contributory to resistance. Herein, we report that APOBEC3 mutagenesis driven by APOBEC3A (A3A) and APOBEC3B (A3B) facilitates breast cancer evolution independent of treatment exposure, leading to resistance against a diverse range of drugs through the induction of APOBEC3-class alterations in characteristic resistance-associated genes. The results highlight APOBEC3 mutagenesis as a biomarker for distinct resistance trajectories, suggesting alternative approaches to target these evolvable cancers.

## Results

### APOBEC3 mutagenesis in metastatic and treatment-resistant breast cancers

**Fig. 1:**
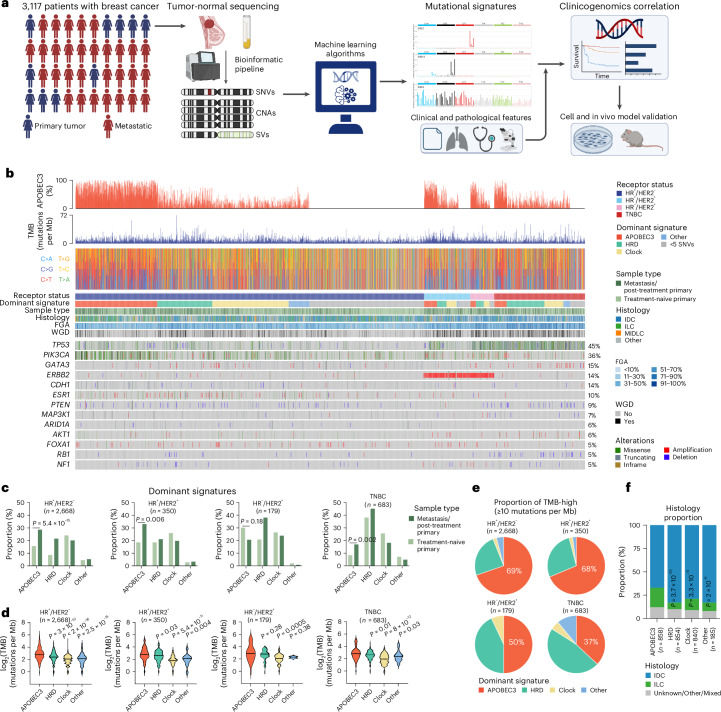
APOBEC3 mutational signatures are prevalent in breast cancers. **a**, Schematic of analysis pipeline of MSK-IMPACT breast cancer cohort. **b**, Summary of genomic characteristics of the clinical cohort demonstrating percentage contribution of APOBEC3 mutational signature (first panel), TMB (second panel), SNV change (third panel) and OncoPrint of select genes in samples. **c**, Barplots displaying the proportion of samples with indicated dominant mutational signature categorized by sample type and receptor status. Groups were compared using the two-tailed Pearsonʼs chi-squared test. **d**, Violin plots representing TMB in samples categorized by receptor status. Groups were compared with APOBEC3-dominant samples using the two-tailed Wilcoxon test. **e**, Proportion of TMB-high samples with different dominant mutational signatures categorized by receptor status. **f**, Proportion of samples categorized by dominant mutational signature and histology. Groups were compared with APOBEC3-dominant samples using the two-tailed Pearson’s chi-squared test. IDC, invasive ductal carcinoma; ILC, invasive lobular carcinoma; MIDLC, mixed invasive ductolobular breast cancer. Panel **a** created using BioRender.com. Source data

**Extended Data Fig. 1:**
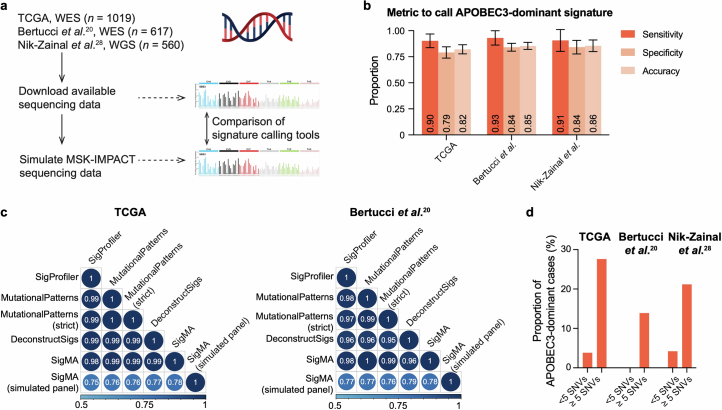
Evaluation of *SigMA* as a tool to assess dominant APOBEC3 mutational signature. (**a**) Schematic of the mutational signature analysis pipeline from publicly available datasets. (**b**) Barplots displaying the sensitivity, specificity, and accuracy of *SigMA* to call APOBEC3 as a dominant signature in the indicated datasets. (**c**) Concordance between the highlighted signature calling tools and *SigMA* in computing mutational signatures from the TCGA and Bertucci et al. datasets. (**d**) Barplots representing the proportion of APOBEC3-dominant samples in the indicated datasets categorized as <5 single nucleotide variants (SNVs) or ≥5 SNVs.

To characterize the mutational landscape of treatment-sensitive and -resistant breast cancers, we leveraged a cohort including over 5,000 cases previously subjected to paired tumor-normal sequencing by the Memorial Sloan Kettering-Integrated Mutation Profiling of Actionable Cancer Targets (MSK-IMPACT) assay^23^. After excluding samples with sequencing-estimated low tumor purity (Methods), 3,880 high-quality samples from 3,117 patients were analyzed (Fig. 1a). This cohort constitutes a large collection of genomically profiled breast cancers coupled with detailed clinical annotation, including treatment and follow-up information, and is representative of the clinical diversity of the disease (Supplementary Table 1). We utilized the single multivariate analysis (SigMA) tool^24^ to deconvolute mutational signatures and assess their contribution to clinical characteristics and outcomes. SigMA has been validated previously for evaluating mutational processes, including homologous recombination deficiency (HRD), in solid tumors^25–27^. To benchmark its validity and fidelity to evaluate APOBEC3 mutational signatures in breast cancers assessed using a targeted sequencing panel, we downsampled publicly available whole exome sequencing (WES) data, including primary breast cancers from The Cancer Genome Atlas (TCGA) (*n* = 1,019) and MBCs from Bertucci et al. (*n* = 617)^20^ as well as whole-genome sequencing (WGS) data of primary breast cancers from Nik-Zainal et al. (*n* = 560)^28^ to the genomic footprint of the MSK-IMPACT panel. We compared the results of SigMA with the dominant signatures called on the WES and WGS data using several analytic tools^29–31^ (Extended Data Fig. 1a). SigMA displayed high sensitivity, specificity and accuracy in detecting APOBEC3 as the dominant signature (Extended Data Fig. 1b). In addition to detecting APOBEC3 as the dominant signature, correlations for exposures calculated from simulated panels using SigMA and WES ranged from 0.75 to 0.79 (Extended Data Fig. 1c and Supplementary Table 2). Finally, considering that SigMA requires at least five single nucleotide variants (SNVs) as input to assess mutational signatures from targeted sequencing, we found that only 0–4% of samples in the three analyzed WES/WGS datasets had fewer than five SNVs (Extended Data Fig. 1d) indicating the ability of SigMA to process most samples from such datasets.

**Extended Data Fig. 2:**
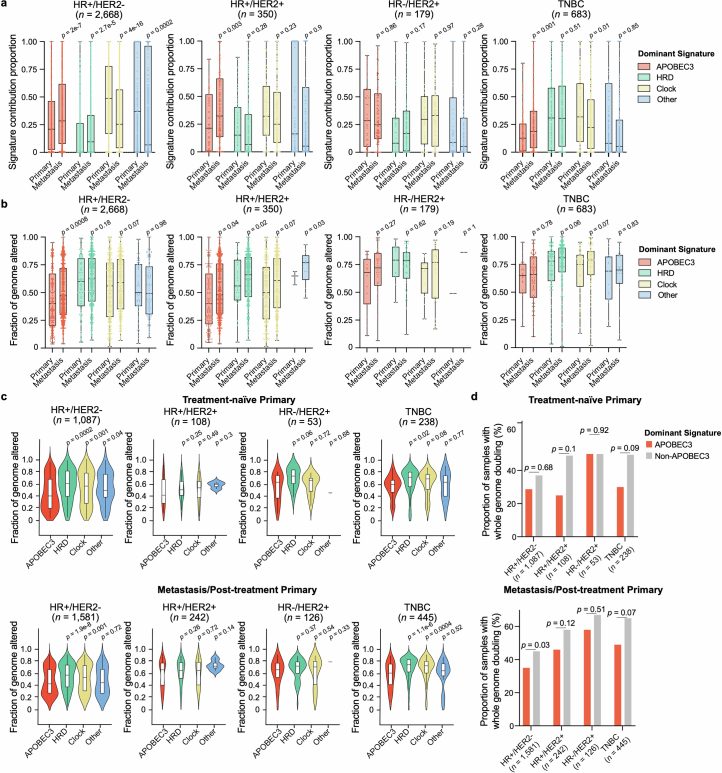
Characterization of APOBEC3 mutational signatures in breast cancers. (**a**) Box plots showing the contribution of APOBEC3, HRD, Clock and Other mutational signatures in primary and metastasis samples categorized according to the receptor status. The primary and metastasis groups were compared using two-tailed Wilcoxon test. (**b**) Box plots displaying the fraction of genome altered (FGA) in primary and metastasis samples categorized according to the dominant mutational signature and receptor status. The primary and metastasis groups were compared using two-tailed Wilcoxon test. (**c**) Violin plots displaying the FGA in primary (top panel) and metastatic (bottom panel) patient samples categorized according to the dominant mutational signature and receptor status. The groups were compared using two-tailed Wilcoxon test. (**d**) Barplots representing the proportion of primary (top panel) and metastatic (bottom panel) patient samples with whole genome doubling (WGD) categorized according to the receptor status. The groups were compared using two-tailed Wilcoxon test.

Examining the 3,880 tumor samples with SigMA, the most prevalent, dominant mutational process was APOBEC3, consistent with previous WGS and WES studies^13,20^ (Fig. 1b). APOBEC3-dominant signature was found in 15.7% and 28.7% of primary and metastatic hormone receptor (HR) positive (HR^+^)/HER2^−^, 18.5% and 33.1% of primary and metastatic HR^+^/HER2^+^, 30.2% and 20.6% of primary and metastatic HR^−^/HER2^+^ and 8.4% and 16.9% of primary and metastatic triple-negative breast cancers (TNBC) (Fig. 1c), respectively. APOBEC3 exposures were significantly higher in HR^+^ and TNBC MBCs than in unmatched primary tumors (Extended Data Fig. 2a), suggesting a link to poor clinical outcomes. We next assessed the relationship between APOBEC3-dominant signature and genomic instability markers such as tumor mutational burden (TMB), fraction of genome altered (FGA) and whole-genome doubling (WGD). APOBEC3-dominant HR^+^ MBC had significantly higher FGA compared with primary breast cancers of the same subtype (Extended Data Fig. 2b), suggesting greater APOBEC3-mediated genomic instability in the metastatic setting. Conversely, APOBEC3-dominant HR^+^/HER2^−^ tumors displayed significantly lower median FGA compared with non-APOBEC3 tumors independent of sample type (Extended Data Fig. 2c), with a similar trend in metastatic TNBC. The proportion of WGD was instead lower in APOBEC3-dominant HR^+^/HER2^−^ MBCs compared with non-APOBEC3 tumors (Extended Data Fig. 2d). APOBEC3-dominant HR^+^ and TNBCs exhibited significantly higher median TMB than those with other mutational processes (Fig. 1d), underscoring the distinct impact of APOBEC3 mutagenesis. For MBCs with TMB of at least ten mutations per megabase, representing the current indication for the tumor agnostic use of anti-programmed death-1 (anti-PD-1) immunotherapy^32^, APOBEC3 constituted the dominant mutational process in most HR^+^/HER2^−^ and HR^+^/HER2^+^ tumors (Fig. 1e). In terms of standard clinical characteristics, groups of APOBEC3-dominant and other dominant signatures were similar (Supplementary Table 3). However, in terms of tumor histology, invasive lobular breast cancers (*n* = 489) were associated more frequently with APOBEC3 mutational signature compared with invasive ductal and other histology types regardless of the sample type (Fig. 1f), consistent with previous evidence^33^.

### APOBEC3 enzymes induce genomic alterations

**Fig. 2:**
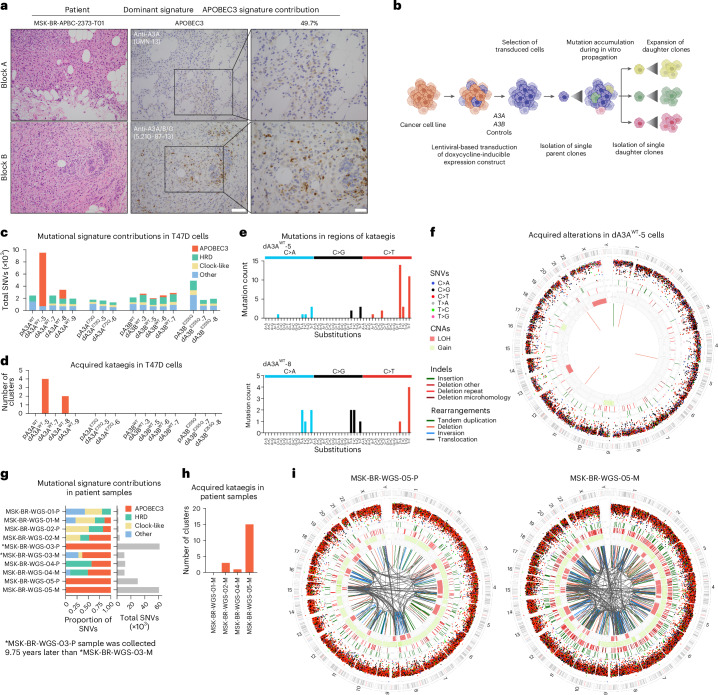
APOBEC3 enzymes induce APOBEC3 mutational processes. **a**, Hematoxylin and eosin staining and immunohistochemical images displaying A3A and A3A/B/G staining in an APOBEC3-dominant patient sample. Scale bars, 50 µm (middle panel) and 20 µm (right panel). In total, *n* = 130 tissue samples were stained. **b**, Schematic of experimental design to investigate ongoing mutational processes in cells overexpressing WT A3A, A3B or their catalytic mutant controls. **c**, Mutational signature contribution of acquired SNVs in the indicated samples. **d**, Barplots representing number of clusters with acquired regions of kataegis in samples from **c**. **e**, Substitution profile of dA3A^WT^-5 and dA3A^WT^-8 in the kataegis regions. **f**, Circos plot representing acquired SNVs, indels, CNAs and structural rearrangements in dA3A^WT^-5 cells. **g**, Mutational signature contribution and number of SNVs from WGS of five paired primary/metastatic patient samples. **h**, Barplots representing number of clusters with acquired regions of kataegis in the indicated metastatic patient samples. **i**, Circos plots of samples MSK-BR-WGS-05-P and MSK-BR-WGS-05-M. d, daughter; p, parent.

**Extended Data Fig. 3:**
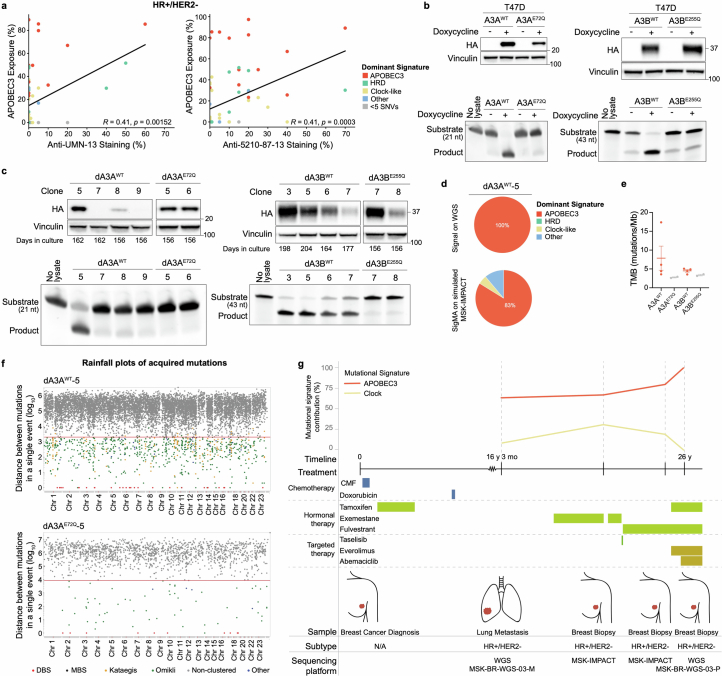
A3A and A3B induce APOBEC3 mutagenesis. (**a**) Scatter plot displaying the correlation between APOBEC3 signature contribution and immunohistochemistry (IHC) staining (in percentage of positive cells) of anti-UMN-13 (left panel) and anti-5210-87-13 (right panel) antibodies in HR+/HER2- breast cancer samples categorized according to the dominant mutational signatures. (**b**) Immunoblots of T47D cells stably transduced with doxycycline-inducible HA-tagged A3A^WT^, A3A^E72Q^, A3B^WT^ or A3B^E255Q^ (top panel). Vinculin was used as a loading control. Single-stranded DNA (ssDNA) deaminase activity of T47D A3A^WT^, A3A^E72Q^, A3B^WT^ or A3B^E255Q^ cells (lower panel). The cells were treated with or without 0.1 µg/mL doxycycline for 48 h before harvesting. (**c**) Western blot (top panel) and ssDNA deaminase activity (lower panel) of the indicated T47D daughter clones of A3A^WT^, A3A^E72Q^, A3B^WT^ or A3B^E255Q^ cells. Vinculin was used as a loading control. The cells were treated with 0.1 µg/mL doxycycline for the indicated number of days. (**d**) Pie charts representing the contribution of mutational signatures in sample dA3A^WT^-5 calculated from the WGS using Signal or the simulated MSK-IMPACT panel using *SigMA*. (**e**) Tumor mutational burden (TMB) in the indicated samples (median TMB 5.25 for A3A^WT^ vs 2.75 for A3A^E72Q^, 4.67 for A3B^WT^ vs 3.36 for A3B^E255Q^). (**f**) Rainfall plots displaying non-clustered (grey) and clustered (colored) mutations in samples dA3A^WT^-5 and dA3A^E72Q^-5. (**g**) Schematic of treatment timeline of MSK-BR-WGS-03. Source data

Out of the 11 APOBEC family members, A3A and A3B have emerged as the main drivers of APOBEC3 mutagenesis^34–38^. To assess their expression in breast cancers harboring APOBEC3 mutational signatures, we performed immunohistochemistry (IHC) on 130 tissue samples using an A3A-specific antibody^39^ or an antibody that detects A3A/B/G^40^ (Fig. 2a). We detected weak to strong protein expression in 17 of 20 APOBEC3-dominant samples with the A3A/B/G antibody and 8 of 20 samples with the A3A-specific antibody, consistent with the reportedly weaker expression of A3A^41,42^. We also observed a weak to moderate correlation between APOBEC3 signature exposure and IHC-based expression in HR^+^/HER2^−^ samples (Extended Data Fig. 3a). Given the known cell-cycle-dependent or episodic expression of APOBEC3 proteins^43–45^, and because mutational signatures reflect accumulation of genomic changes over the lifetime of a cell, these findings cannot establish whether high levels of expression are necessary for signature manifestation. Our findings suggest that mutational signatures might be a more robust method to detect tumors with APOBEC3 activity.

To evaluate directly whether A3A or A3B can cause APOBEC3 mutagenesis in breast cancer cells, we overexpressed HA-tagged wild-type (WT) enzymes and their catalytically inactive mutants (A3A^E72Q^ or A3B^E255Q^) in ER-positive breast cancer cells lacking endogenous APOBEC3 activity^46^ using a doxycycline-inducible system (Extended Data Fig. 3b). We observed in vitro DNA deaminase activity with overexpression of both WT enzymes but not the mutant controls. To assess the acquired alterations upon A3A or A3B overexpression, we isolated single-cell parent clones and exposed them to doxycycline for 72 days to drive mutagenesis followed by expansion of several single-cell daughter clones (Fig. 2b). Interestingly, two out of four A3A WT-expressing daughter clones lost protein expression and deaminase activity (Extended Data Fig. 3c). Similar negative selection against A3A expression has been reported earlier^34,39^. We performed WGS of parent and daughter clones and analyzed acquired alterations. The lack of endogenous APOBEC3 activity in the model was confirmed by the absence of acquired APOBEC3 signature in the parental cells before doxycycline treatment (Fig. 2c). Among the daughter cells that maintained protein expression, A3A and A3B WT cells accumulated APOBEC3-context mutations (41.3–92.5% of the total SNVs in A3A and 6.7–12.5% in A3B), whereas the mutant cells did not (Fig. 2c and Supplementary Table 4). The daughter cells that lost A3A expression and activity did not display any acquired APOBEC3 exposure, further confirming the necessity of cytidine deaminase activity for signature accumulation. As an additional confirmation of the proficiency of SigMA to detect APOBEC3 signatures in a controlled experiment, we simulated the WGS of cell lines to the MSK-IMPACT panel and used SigMA to detect mutational signatures. Among the samples with at least five SNVs, SigMA successfully classified dA3A^WT^-5 as APOBEC3-dominant, confirming the high performance using the WES and WGS datasets (Extended Data Fig. 3d). The WT cells exhibited higher TMB compared with mutant controls (Extended Data Fig. 3e). Cells with APOBEC3 as the dominant signature—APOBEC3-positive cells—also accumulated increased clustered mutations characterized as kataegis^47^ and omikli^48^, which have been linked previously to APOBEC3 activity (Fig. 2d and Extended Data Fig. 3f). Most mutations in the regions of kataegis were substitutions in the APOBEC3-enzyme-recognized TCN motifs, where C is the target cytosine (Fig. 2e).

In addition to single nucleotide changes, comparative analyses revealed other acquired genomic alterations, including insertions and deletions (indels), copy number alterations (CNAs) and structural variations (SVs) (Fig. 2f). To assess these non-SNV alterations in the clinical cohort, we performed WGS of five pairs of primary and metastatic patient samples (Fig. 2g and Supplementary Table 5), reflecting breast cancers with varying levels of APOBEC3 signatures. Some metastatic samples showed acquired APOBEC3 signature indicating APOBEC3 mutagenic activity during treatment resistance. As an example, we present the progressive evolution of an HR^+^/HER2^−^ tumor to 100% APOBEC3 exposure over more than 20 years of clinical history and therapeutic pressure of several lines of treatment, including endocrine therapies, CDK4/6i, and PI3K-pathway inhibitors (paired samples from MSK-BR-WGS-03; Extended Data Fig. 3g). Similar to cell lines, APOBEC3-positive samples acquired clustered mutations characterized as kataegis (Fig. 2h). These samples also exhibited substantial genomic alterations including indels, CNAs, SVs and chromothripsis—a hallmark of APOBEC3 activity^25,49^ (Fig. 2i). This phenomenon, characterized by clustered chromosomal rearrangements in a single event causing complex genomic alterations, is observed in sample MSK-BR-WGS-05-M on chromosomes 15, 8 and X. Given the similarities with APOBEC3-positive cells, we conclude that A3A and A3B overexpression phenocopies APOBEC3-driven tumors in a deamination-dependent manner.

### APOBEC3 activity promotes therapy resistance

**Fig. 3:**
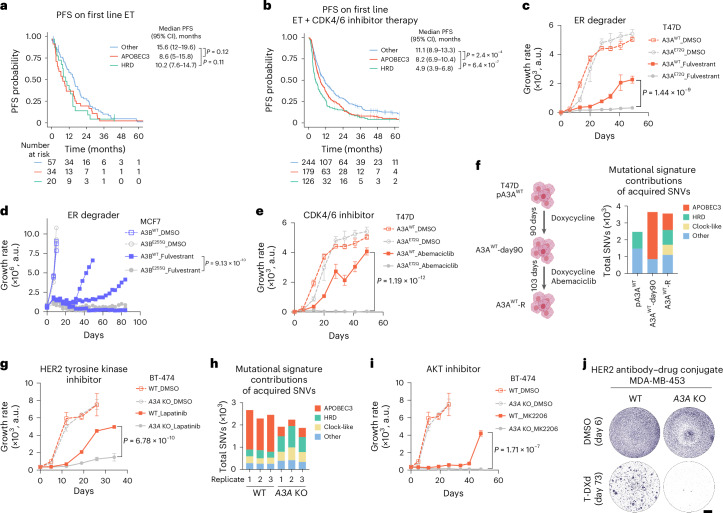
APOBEC3 mutagenesis promotes therapeutic resistance in breast cancers. **a**,**b**, Kaplan–Meier curves displaying PFS probability of patients with HR^+^/HER2^−^ MBCs treated with ET as single agent (PFS 8.6 versus 15.6 months in APOBEC3-dominant tumors and tumors with Other dominant signatures, respectively; hazard ratio, 1.4; 95% CI, 0.9–2.2; *P* = 0.12) (**a**) or in combination with CDK4/6 inhibition (hazard ratio, 1.5; 95% CI, 1.2–1.8; *P* = 2.4 × 10^−4^ for APOBEC3-dominant versus Others and hazard ratio, 1.8; 95% CI, 1.4–2.2; *P* = 6.4 × 10^−7^ for HRD-dominant versus Others) (**b**). Patients were categorized according to the dominant mutational signatures of the biopsy obtained before start of treatment. Groups were compared using log rank test. **c**, Growth curves of T47D A3A^WT^ and A3A^E72Q^ cells treated with DMSO or fulvestrant (10 nM). Data represent mean ± s.d. of three replicates. The groups were compared using two-way ANOVA test. **d**, Growth curves of MCF7 A3B^WT^ and A3B^E255Q^ cells treated with DMSO or fulvestrant (10 nM). Data are represented as individual replicates (*n* = 3). Groups were compared using two-tailed Mann–Whitney *U* test. **e**, Growth curves of T47D A3A^WT^ and A3A^E72Q^ cells treated with DMSO or abemaciclib (500 nM). Data represent mean ± s.d. of three replicates. Groups were compared using two-way ANOVA test. **f**, Schematic showing the timeline of generation of abemaciclib-resistant T47D A3A^WT^ (A3A^WT^-R) cells (left panel). Mutational signature contribution of acquired SNVs in the samples indicated (right panel). **g**, Growth curves of BT-474 WT and *A3A* KO cells treated with DMSO or lapatinib (20 nM). Data represent mean ± s.d. of three replicates. Groups were compared using two-way ANOVA test. **h**, Mutational signature contribution of acquired SNVs in the samples indicated. **i**, Growth curves of BT-474 WT and *A3A* KO cells treated with DMSO or MK2206 (100 nM). Data represent mean ± s.d. of three replicates. Groups were compared using two-way ANOVA test. **j**, Crystal violet staining of MDA-MB-453 WT and *A3A* KO cells treated with DMSO (for 6 days) or T-DXd (100 ng ml^−1^, for 73 days). Images are representative of *n* = 3 replicates. Scale bar, 200 µm. a.u., arbitrary units.

**Extended Data Fig. 4:**
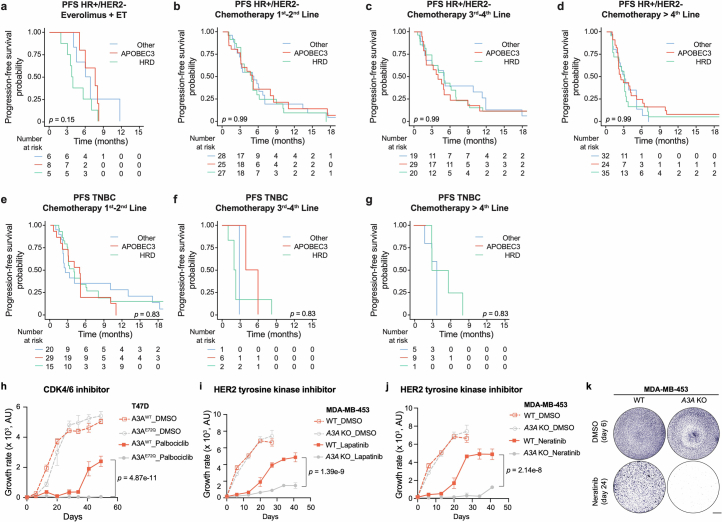
APOBEC3 activity drives therapeutic resistance in breast cancers. (**a-g**) Kaplan-Meier curves displaying progression-free survival probability of patients with (**a**) HR+/HER2- metastatic breast cancers treated with everolimus and endocrine therapy, (**b**) HR+/HER2- metastatic breast cancers treated with chemotherapy in 1^st^-2^nd^ line, (**c**) HR+/HER2- metastatic breast cancers treated with chemotherapy in 3^rd^-4^th^ line, (**d**) HR+/HER2- metastatic breast cancers treated with chemotherapy in >4^th^ line, (**e**) TNBC metastatic breast cancers treated with chemotherapy in 1^st^-2^nd^ line, (**f**) TNBC metastatic breast cancers treated with chemotherapy in 3^rd^-4^th^ line or (**g**) TNBC metastatic breast cancers treated with chemotherapy in >4^th^ line. The patients are categorized according to the dominant mutational signatures of the biopsy obtained prior to treatment start. The groups were compared using two-sided log-rank test. (**h**) Growth curves of T47D A3A^WT^ and A3A^E72Q^ cells treated with DMSO or palbociclib (500 nM). Data are represented as mean ± SD of three replicates. The groups were compared using two-way ANOVA test. AU is abstract unit. (**i, j**) Growth curves of MDA-MB-453 WT and *A3A* KO cells treated with DMSO or (**i**) lapatinib (2 µM) or (**j**) neratinib (100 nM). Data are represented as mean ± SD of three replicates. The groups were compared using two-way ANOVA test. (**k**) Crystal violet staining of MDA-MB-453 WT and *A3A* KO cells treated with DMSO (for 6 days) or neratinib (100 nM, for 24 days). Images are representative of *n* = 3 replicates. Scale bar = 200 µm.

To investigate the relationship between APOBEC3 signature and therapy resistance, we leveraged the clinical annotations linked to our cohort and assessed outcomes of APOBEC3-dominant cases on various therapies. To avoid inconsistencies related to changes of APOBEC3 mutagenesis over time, only patients with biopsies acquired directly before the first dose of the indicated treatment (±30 days) were included. In HR^+^/HER2^−^ MBC treated with first-line endocrine monotherapy (*n* = 111), APOBEC3-dominant tumors exhibited numerically shorter median progression-free survival (PFS) compared with tumors with other dominant signatures (Fig. 3a). For first-line therapy with CDK4/6i plus ET (*n* = 549), APOBEC3- and HRD-dominant MBC were associated independently with lower median PFS compared with other cancers regardless of ET partner and line of therapy (Fig. 3b) suggesting increased genomic instability confers resistance to standard frontline therapy in HR^+^/HER2^−^ MBC. To further assess the clinical impact of APOBEC3 mutagenesis in MBC, we compared outcomes in patients receiving other treatments, including everolimus-based combinations for HR^+^/HER2^−^ tumors and different chemotherapy regimens for HR^+^/HER2^−^ and TNBC subtypes (Extended Data Fig. 4a–g). Our analysis in these smaller cohorts did not reveal a significant effect of mutational processes on treatment response.

To assess the causal relationship between therapy resistance and APOBEC3 mutagenesis, we employed our long-term doxycycline-treated APOBEC3-positive and APOBEC3-negative models. The growth rate of APOBEC3-positive A3A^WT^ and APOBEC3-negative A3A^E72Q^ cells was comparable under dimethylsulfoxide (DMSO) treatment (Fig. 3c and Supplementary Table 6). When exposed to the SERD fulvestrant, A3A^WT^ cells acquired resistance significantly faster than A3A^E72Q^ cells. In a similar experiment with the weaker mutator A3B, two out of three replicates of A3B^WT^ cells acquired resistance to fulvestrant, whereas none of the three A3B^E255Q^ replicates developed resistance after 84 days of continuous drug exposure (Fig. 3d). A3A WT overexpression also led to a selective growth advantage of A3A^WT^ cells on treatment with the CDK4/6i abemaciclib (Fig. 3e) and palbociclib (Extended Data Fig. 4h). Based on analysis of the acquired alterations using WGS, A3A^WT^ cells continued to accumulate APOBEC3 signature mutations during resistance development (Fig. 3f), further implying a role of APOBEC3 activity in driving resistance.

We next tested the effect of endogenous APOBEC3 enzymes in facilitating resistance. For this, we used the HER2^+^ models with endogenously active APOBEC3 mutagenesis that is predominantly driven by A3A^36^. Similar to our ER-positive models, the APOBEC3-positive BT-474 WT cells acquired resistance to the tyrosine kinase inhibitor lapatinib significantly faster than the APOBEC3-negative *A3A* knockout (KO) cells (Fig. 3g). BT-474 cells also maintained endogenous APOBEC3 activity during treatment pressure (Fig. 3h), confirmed by WGS of pre- and post-treatment cells. We also observed a significant growth advantage of MDA-MB-453 WT cells with tyrosine kinase inhibitors lapatinib and neratinib (Extended Data Fig. 4i–k). WT cells selectively gained resistance to other anti-HER2 therapies including an inhibitor of the downstream target AKT kinase (AKT), MK2206 (Fig. 3i) or the antibody–drug conjugate, T-DXd (Fig. 3j). Overall, our data demonstrate that APOBEC3 activity can promote therapy resistance in several contexts.

### Mechanisms of APOBEC3-mediated resistance

**Fig. 4:**
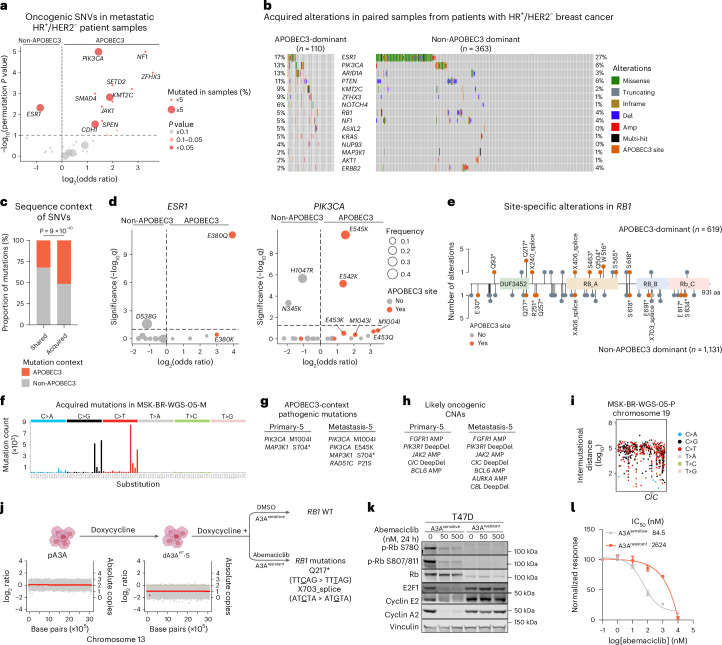
APOBEC3-class alterations drive therapeutic resistance in breast cancers. **a**, Volcano plot displaying enrichment of genes in metastatic HR^+^/HER2^−^ samples categorized according to the dominant mutational signature. Odds ratios (log_2_ transformed) were computed by logistic regression (two-sided) with *P* values (−log_10_ transformed) corrected by permutation test. Genes are represented as circles, color coded and sized according to the legend. **b**, OncoPrint of acquired alterations in HR^+^/HER2^−^ tumor samples categorized according to the dominant mutational signature. **c**, Barplots representing proportion of shared or acquired SNVs categorized as APOBEC3-context and non-APOBEC3-context SNVs. Groups were compared using the two-sided Fisher’s exact test. **d**, Volcano plots depicting site-specific enrichment of *ESR1* (left panel) and *PIK3CA* (right panel) categorized according to the dominant mutational signature. Odds ratios (log_2_ transformed) were computed by logistic regression with *P* values (−log_10_ transformed) corrected for FDR by Benjamini–Hochberg method. Genes are represented as circles, color coded and sized according to the legend. **e**, Site-specific enrichment of alterations in *RB1* gene in HR^+^/HER2^−^ tumor samples. **f**, Mutation spectrum of acquired SNVs in MSK-BR-WGS-05-M. **g**, Pathogenic mutations with APOBEC3-context substitutions in samples MSK-BR-WGS-05-P and MSK-BR-WGS-05-M. Asterisk indicates nonsense mutations. **h**, Likely oncogenic CNAs in samples MSK-BR-WGS-05-P and MSK-BR-WGS-05-M. **i**, Rainfall plot displaying intermutational distance between SNVs in chromosome 19 of sample MSK-BR-WGS-05-P. **j**, FACETS plot showing LOH of chromosome 13 in dA3A^WT^-5 and acquired SNVs in *RB1* after DMSO (A3A^sensitive^) or abemaciclib (1 µM) (A3A^resistant^) treatment. Asterisk indicates nonsense mutations. **k**, Immunoblots displaying changes in cell-cycle regulatory proteins in A3A^sensitive^ and A3A^resistant^ cells treated with DMSO or indicated doses of abemaciclib for 24 h. Vinculin was used as a loading control. Immunoblots are representative of *n* = 3 independent experiments. **l**, Inhibition of proliferation of A3A^sensitive^ and A3A^resistant^ cells treated with increasing concentrations of abemaciclib. Data represent mean ± s.d. of three replicates and are normalized to the DMSO control. Source data

**Extended Data Fig. 5:**
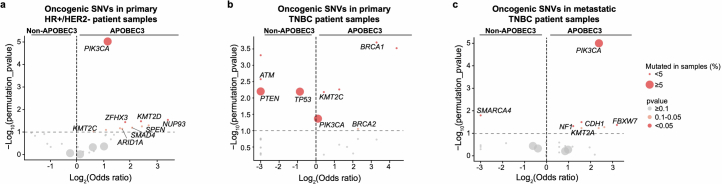
Gene enrichment in breast cancer samples. Volcano plots depicting enrichment of genes in (a) primary HR+/HER2-, (b) primary TNBC or (c) metastatic TNBC samples categorized according to the dominant mutational signature.

To understand mechanistically how APOBEC3 mutagenesis drives resistance in breast cancers, we assessed whether alterations in genes linked to therapy resistance were induced specifically by APOBEC3 mutagenesis. We first conducted a gene enrichment analysis using the MSK-IMPACT breast cancer cohort. To address potential biases from the APOBEC3-induced hypermutator phenotype, we applied a permutation test (Methods). We observed a significant enrichment of oncogenic mutations affecting *PIK3CA*, *CDH1* and *KMT2C* (*P*_permutation_ < 0.1, mutated in >5% samples) in metastatic/post-treatment HR^+^/HER2^−^ APOBEC3-dominant tumors (Fig. 4a and Supplementary Table 7). Similarly, APOBEC3-dominant, treatment-naive HR^+^/HER2^−^ or all TNBC samples also exhibited an enrichment of *PIK3CA* variants compared with non-APOBEC3-dominant tumors (Extended Data Fig. 5a–c). Oncogenic mutations in other resistance-associated genes such as *NF1* and *ZFHX3* were also enriched in APOBEC3-dominant samples, albeit at lower frequencies (2.8% for *NF1* and 1.1% for *ZFHX3*). We next examined acquired alterations in patients with HR^+^/HER2^−^ breast cancer who had several tumor samples collected over time (*n* = 449 with two samples, *n* = 43 with at least three samples), focusing on APOBEC3-context mutations in samples with evidence of APOBEC3 activity. After de novo genotyping of somatic mutations (Methods), we observed an enrichment of APOBEC3-context, acquired alterations in genes encoding transcription factors linked previously to ET resistance, such as *ARID1A* and *ZFHX3* (refs. ^50,51^). These alterations were enriched in treatment-resistant tumors with dominant APOBEC3 signature compared with non-APOBEC3-dominant samples (*q* = 0.08 for *ARID1A*, *q* = 0.08 for *ZFHX3*) (Fig. 4b). The proportions of acquired alterations in key genes of the PI3K/AKT pathway, including *PIK3CA* (*q* = 0.4) and *PTEN* (*q* = 0.5), and other resistance-linked genes such as *KMT2C* (*q* = 0.2) were also numerically higher in APOBEC3-dominant, therapy-resistant samples. We found that the proportion of APOBEC3-context mutations exclusive (acquired) to later-stage samples was notably higher than those shared with earlier or treatment-naive samples from the same patient (51.9% versus 32.3%; Fig. 4c) suggesting an active role of APOBEC3 mutagenesis in driving the resistance-linked mutations described above.

**Extended Data Fig. 6:**
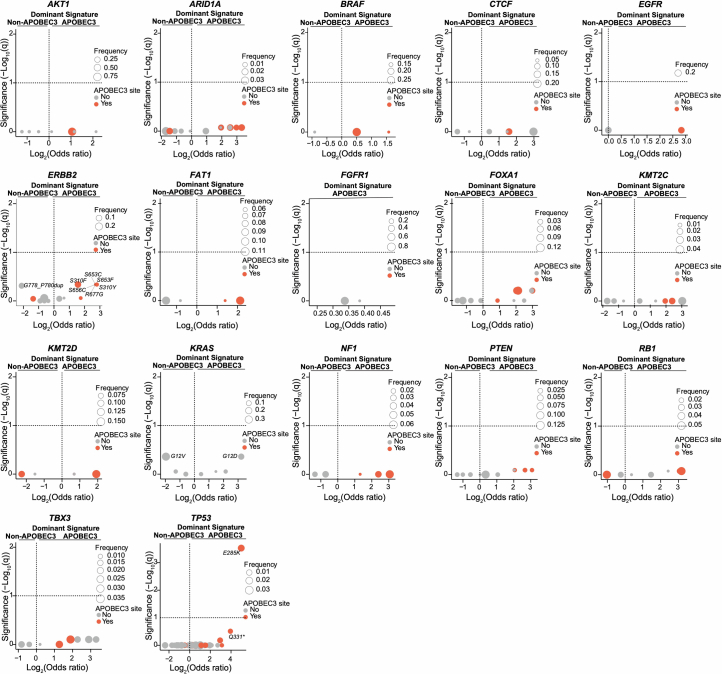
Site-specific enrichment in HR+/HER2- breast cancer samples. Volcano plots depicting site-specific enrichment of the indicated genes categorized according to the dominant mutational signature.

Alterations in *ESR1* are the most frequent acquired resistance alterations in MBC. In our paired analysis of HR^+^/HER2^−^ samples, we observed that 17% of APOBEC3-dominant samples presented with *ESR1* alterations compared with 27% non-APOBEC3-dominant samples (*q* = 0.4; Fig. 4b). To explore this further, we examined site-specific gene enrichment of all genes included in the MSK-IMPACT panel that were previously linked to ET resistance (Extended Data Fig. 6). Strikingly, *ESR1* E380Q mutations—an APOBEC3-context substitution—were enriched specifically in APOBEC3-dominant, HR^+^/HER2^−^ post-treatment samples (*q* = 7.8 × 10^−12^; Fig. 4d). By contrast, highly activating *ESR1* mutations in the helix 11–12 loop (L536X, Y537X and D538X, none of which are APOBEC3-context substitutions) were rare (8%) in APOBEC3-dominant samples, suggesting that APOBEC3-dominant tumors might not dominantly reactivate ER as the mechanism of endocrine resistance. Among other common mutations, *PIK3CA* hotspot mutations, such as E545K (*P* = 2 × 10^−10^) and E542K (*P* = 4.5 × 10^−6^), which are APOBEC3-context mutations, were detected predominantly in APOBEC3-dominant HR^+^/HER2^−^ post-treatment samples (Fig. 4d), confirming the enrichment of *PIK3CA* helical domain mutations in APOBEC3-dominant breast cancers^52^. These findings suggest that APOBEC3 contributes to site-specific resistance-associated alterations, but not necessarily all resistance-causing changes, also exemplified by those observed for the tumor suppressor *RB1* (Fig. 4e).

**Extended Data Fig. 7:**
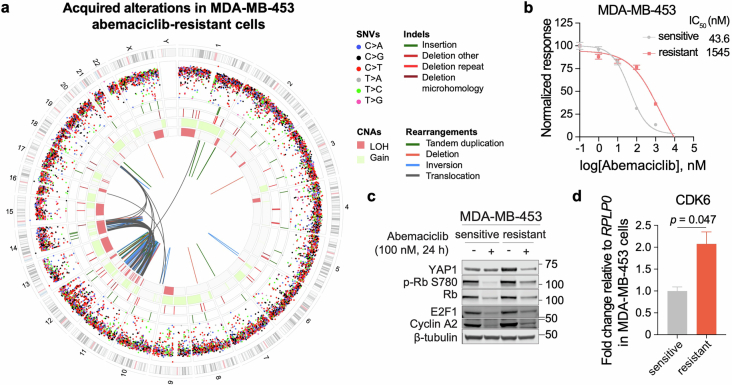
A3A-driven abemaciclib resistance in MDA-MB-453 cells. (**a**) Circos plot displaying acquired changes in MDA-MB-453 WT cells upon abemaciclib treatment. (**b**) Inhibition of proliferation of MDA-MB-453 WT sensitive and resistant cells treated with increasing concentrations of abemaciclib. Data are represented as mean ± SD of three replicates and are normalized to the DMSO control. (**c**) Immunoblots displaying changes in cell cycle regulatory proteins in MDA-MB-453 WT sensitive and resistant cells treated with DMSO or abemaciclib (100 nM) for 24 h. Vinculin was used as a loading control. (**d**) Bar plot displaying the fold change in *CDK6* mRNA expression in MDA-MB-453 WT sensitive and resistant cells. Data are represented as mean ± SD of three replicates. Source data

As a salient example of involvement of APOBEC3 mutagenesis in acquired resistance to ET, we explored in depth the WGS of patient MSK-BR-WGS-05 (Fig. 2g) who received ET including the selective ER modulator tamoxifen and the aromatase inhibitor letrozole. Of the acquired mutations, 95% were assigned to APOBEC3 signature (Fig. 4f), implying that APOBEC3 mutational processes were active at some point during progression and resistance development. The cancer specifically acquired APOBEC3-context pathogenic mutations in *PIK3CA* (E545K) and *RAD51C* (P21S) (Fig. 4g). In terms of CNAs, the cancer maintained the *FGFR1* amplification and *PIK3R1* deep deletion from the primary sample and acquired an amplification in *AURKA* in the metastatic sample (Fig. 4h). We observed a high-density kataegis region in chromosome 19, in the gene *CIC*, which presented a deep deletion in both the primary and the metastatic samples (Fig. 4i). The proximity of such clustered mutations that are highly associated with APOBEC3 activity, hints at a role for APOBEC3 mutagenesis in contributing to some such CNAs in addition to the point mutations. We also detected evidence of such chromosomal rearrangements in our APOBEC3-positive cells tested for therapy resistance. In one case, upon A3A WT overexpression, the T47D daughter cells (dA3A^WT^-5) acquired a loss of heterozygosity (LOH) event in chromosome 13 (Fig. 4j). Similar chromosome 13 LOH was present in three out of six APOBEC3-positive cells but none of the ten APOBEC3-negative cells (*P* = 0.035). When dA3A^WT^-5 cells were exposed to abemaciclib, they selectively acquired APOBEC3-context mutations in *RB1*. Q217* was the SNV observed most frequently in *RB1* in our clinical cohort, enriched in APOBEC3-dominant cases (Fig. 4e). The lone non-APOBEC3-dominant case with this mutation nonetheless exhibited 63.5% of APOBEC3 signature contribution, suggesting a causal role of APOBEC3 mutagenesis in inducing this truncating mutation. These loss-of-function mutations in *RB1* led to a complete loss of RB1 protein expression (Fig. 4k) and insensitivity to abemaciclib. The resistance was also confirmed by an increase of ~31-fold in half maximal inhibitory concentration (IC_50_) values of the resistant cells compared with DMSO-treated cells (Fig. 4l). In another example, MDA-MB-453 WT cells, which exhibit endogenous A3A-driven mutagenesis, developed resistance to abemaciclib through a chromothripsis event targeting the *YAP1* locus on chromosome 11 (Extended Data Fig. 7a,b). This event resulted in elevated YAP1 expression and increased expression of its downstream target gene, CDK6 (Extended Data Fig. 7c,d), which is a known and targetable mechanism of resistance to CDK4/6i^7,53,54^. Overall, these cases illustrate both pre-existence of APOBEC3 mutagenesis before drug exposure, but also the marked accumulation and diversification over time, arguing for APOBEC3 being an active mutagenic process. Together with the clinical observations, our data reveal that APOBEC3 mutagenesis leads specifically to APOBEC3-class alterations that drive therapy resistance and promote lethal outcomes in MBC.

### Targeting APOBEC3-enriched breast cancers

**Fig. 5:**
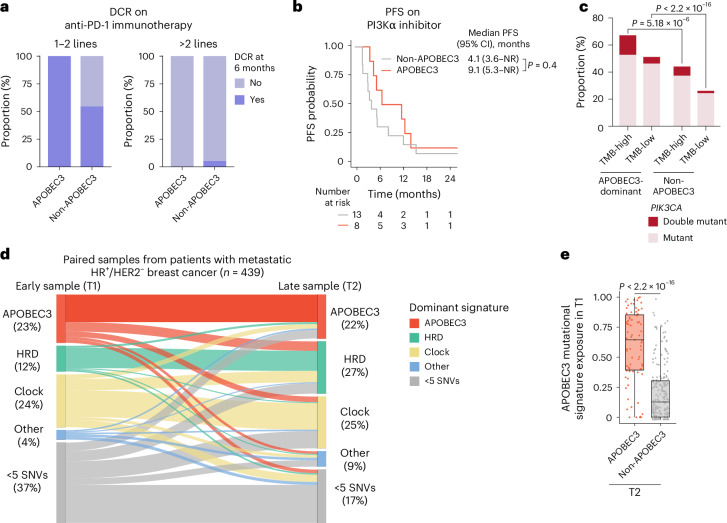
Vulnerabilities of APOBEC3-dominant breast cancers. **a**, Barplots displaying the proportion of APOBEC3-dominant and non-APOBEC3-dominant tumors categorized based on the disease control rate (DCR) on 1–2 lines or >2 lines of anti-PD-1 immunotherapy. **b**, Kaplan–Meier curves displaying PFS probability of patients with HR^+^/HER2^−^ MBCs treated with PI3Kα inhibitor. Patients were categorized according to the dominant mutational signatures of the biopsy obtained before start of treatment. Groups were compared using log rank test. **c**, Barplots representing proportion of *PIK3CA* mutations in APOBEC3-dominant and non-APOBEC3-dominant tumors categorized as TMB-high or TMB-low. Groups were compared using the two-sided Fisher’s exact test. **d**, Sankey plots representing the evolution of early/late paired HR^+^/HER2^−^ patient samples categorized according to the dominant mutational signatures. **e**, Boxplots displaying the mutational signature contribution in paired earlier samples when the later sample is APOBEC3-dominant (*n* = 439). Groups were compared using the two-tailed Wilcoxon test. Boxplots display the first quartile (Q1), median and third quartile (Q3), with whiskers extending to the nearest datapoints within Q1 − 1.5 × IQR and Q3 + 1.5 × IQR.

**Extended Data Fig. 8:**
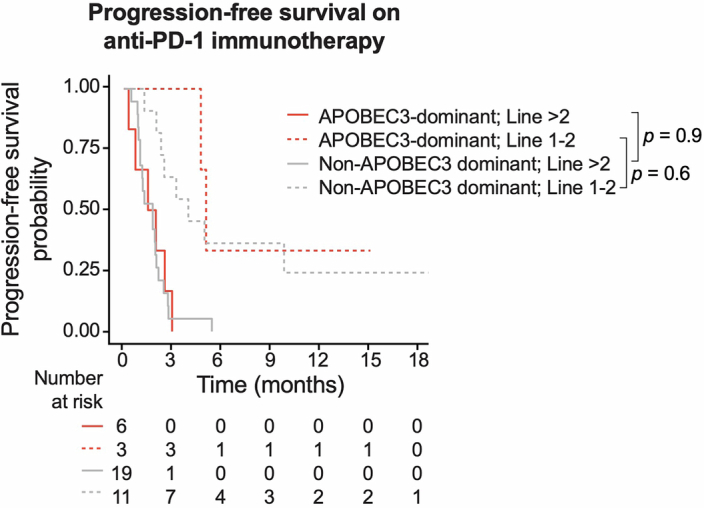
Outcome of HR+/HER2- breast cancers to anti-PD-1 immunotherapy. Kaplan-Meier curves displaying progression-free survival probability of patients with HR+/HER2- metastatic breast cancers treated with anti-PD-1 immunotherapy. The patients are categorized according to the dominant mutational signatures of the biopsy obtained prior to treatment start and the line of treatment. The groups were compared using the two-sided log-rank test.

Our results establish a causal role for APOBEC3 mutagenesis in promoting resistance in HR^+^/HER2^−^ breast cancers, revealing the necessity of improving targeting of APOBEC3-dominant tumors. In the absence of reliable APOBEC3-targeting agents, we explored whether we could exploit the specific biomarkers enriched in APOBEC3-dominant tumors that serve as indications for targeted therapies (*PIK3CA* mutation for PI3Kα-selective inhibitors^55^ and high TMB for anti-PD-1 monoclonal antibodies^56^). We identified 39 TMB-high, HR^+^/HER2^−^ MBC patients who received anti-PD-1 immunotherapy. Although no statistically significant difference in median PFS was observed in APOBEC3-dominant versus non-APOBEC3-dominant tumors (first/second line, 5.1 versus 3.9 months; more than second line, 1.7 versus 1.7 months; Extended Data Fig. 8), patients treated in earlier lines (first/second lines) displayed a numerically longer PFS. Among these (*n* = 14), all patients with APOBEC3-dominant tumors showed disease control (Fig. 5a). We next examined patients who received PI3Kα-selective inhibitors for HR^+^/HER2^−^ MBC. Although numbers were limited to effectively compare PFS based on dominant signatures (fewer than four lines, *n* = 21), several APOBEC3-dominant cases were treated for over 6 months (*n* = 5 of 8), with one patient responding for more than 30 months (Fig. 5b). We found a higher rate of double *PIK3CA* mutations among APOBEC3-dominant cases, regardless of TMB category (Fig. 5c), suggesting that APOBEC3 mutagenesis may promote mutations that sensitize tumors to PI3Kα-selective inhibitors^57^.

Finally, given the potential utility of earlier identification and use of targeted and combination therapies in breast cancer, we explored whether APOBEC3 signatures might be evident in earlier stages using the paired cohort (Fig. 5d). Over 60% of cases with APOBEC3 as the dominant signature at a later timepoint also showed it at an earlier timepoint, with a median exposure of 64% (interquartile range (IQR), 39–85%; Fig. 5e). These findings reveal a persistent role for APOBEC3 mutagenesis in most cases, rather than a de novo acquisition during treatment exposure, and highlight the potential of APOBEC3 signatures to identify tumors at risk for development of treatment resistance.

## Discussion

In this report, we demonstrate that treatment refractory, poor prognosis breast cancers are marked by pervasive APOBEC3 mutagenesis. In patient samples and laboratory models, APOBEC3 enzymes A3A and A3B drive tumor evolution and treatment resistance by inducing APOBEC3-context alterations in resistance-associated genes. Although APOBEC3 activity promotes new, subclonal alterations mediating resistance, evidence for APOBEC3 activity is detectable in pretreatment samples, suggesting APOBEC3 signatures as potential biomarkers and revealing alternatives to targeting these high-risk cancers.

Although identifying specific mutations that mediate therapy resistance in advanced cancers has led to improved second-generation inhibitors or combinations, many cancers evolve to subvert these therapies too. This highlights the need to understand the evolutionary processes enabling resistance. Such insights can identify the cancers at risk for future resistance and inform strategies to overcome it. The widespread prevalence of APOBEC3 mutational signatures in MBC suggested its relevance for tumor evolution, prompting us to examine its specific function in this context.

First, by examining a cohort of patients with detailed treatment histories, we established that APOBEC3 mutagenesis is associated with shorter outcomes on targeted therapies, including antiestrogen monotherapy and antiestrogen plus CDK4/6i combinations among HR^+^ cancers. To evaluate causality of APOBEC3 mutagenesis in resistance, we utilized laboratory models in which APOBEC3 mutagenesis was either introduced or abrogated. In both cases, APOBEC3 mutagenesis reproducibly accelerated the capacity of tumor cells to develop resistance. In some models, this process drove resistance by introducing the same alterations observed in patients with therapy resistance such as *RB1* loss-of-function mutations after exposure to CDK4/6i. Our clinical data also point to resistance alterations (for example, *ESR1* E380Q or *ARID1A* truncating mutations), among APOBEC3-dominant tumors generated in the A3A or A3B context. These findings establish APOBEC3 activity as a key factor in therapy resistance, raising questions about additional types of genomic alterations enriched in APOBEC3-dominant tumors, the role of A3A and/or A3B in these alterations and when these changes occur in disease progression.

The timing of APOBEC3 mutagenesis remains controversial, with evidence for both persistent and staggered events^44^. Moreover, the enrichment of APOBEC3 mutagenesis in MBC compared with primary disease has further raised the question of whether APOBEC3 activity is ‘acquired,’ such as *ESR1* mutations, or is intrinsic to the evolvable nature of these cancers. The ability to profile a large cohort of paired primary and metastatic tumors provides some answers. We identified that 60% of APOBEC3-dominant tumors had APOBEC3 signatures at earlier stages, and 95% showed A3A or A3B protein expression, supporting the idea that APOBEC3 activity exists early on. This is consistent with our finding of APOBEC3 signatures in some pre-invasive breast lesions^33,58^, and is distinct from other cancer types where APOBEC3 activation may be initiated by targeted therapy itself^59,60^. The pre-existence of APOBEC3 in breast cancer establishes the potential for the development of biomarkers that can detect this underlying evolvability before exposure to therapy. Such biomarkers are likely to be the first step toward enabling treatment strategies that overcome resistance and limit the use of unnecessary therapies to those not at risk of such evolution.

There has been limited evaluation of therapies specifically for APOBEC3-dominant cancers. The genomic patterns of APOBEC3-dominant tumors reveal two promising targets. First, APOBEC3-dominant tumors often harbor mutations in the helical domain of *PIK3CA* and double *PIK3CA* mutations, indicating a unique dependence on the PI3K pathway. This suggests a subset uniquely vulnerable to PI3K-targeting therapy in early stages before resistance mutations arise^11,61^. Second, APOBEC3-dominant tumors often display high TMB, an indication for immune checkpoint blockade across solid tumors^32^. Indeed, a subset of patients treated under this indication were APOBEC3-dominant and, among these, several prolonged responses were observed in the metastatic, treatment-refractory setting. The potential for improved efficacy of immunotherapies in early-stage disease raises the possibility of using APOBEC3 as a biomarker where the field currently lacks determinants of benefit^62^. However, even beyond these possibilities, many existing therapies (CDK4/6i, antibody–drug conjugates, and so on) may benefit from signature-based biomarkers that incorporate a cancer’s potential for evolution.

This study has several limitations that will be important to address with future research. First, the clinic-genomic cohort was evaluated by targeted panel sequencing with reduced information than WGS/WES. Although we have taken steps to ensure the fidelity of our findings, the use of WGS will probably enable further understanding of the clinical scope of APOBEC3 mutagenesis. Another limitation is the preponderance of ER-positive tumors in our cohort, reducing our ability to ascertain how APOBEC3 mutagenesis impacts disease progression in other breast cancer subtypes. Moreover, the large proportion of metastatic cases limits analysis of the role of APOBEC3 on relapse of early-stage disease. Larger cohorts of primary cancers with long-term follow-up, including cases that never relapse, are needed to assess the specific contributions of APOBEC3 mutagenesis to metastatic relapse and resistance to adjuvant therapies.

In closing, we find that APOBEC3 mutagenesis represents a highly prevalent driver of genomic instability in breast cancer, contributing specifically to resistance to endocrine and targeted therapies. Our results further demonstrate that APOBEC3 activity can be detected before exposure to therapies in breast cancer, making it a potential biomarker and therapeutic target in this disease.

## Methods

### Study cohort

This study was approved by the Memorial Sloan Kettering Cancer Center (MSKCC) Institutional Review Board (12-245) and all patients provided written informed consent for tumor sequencing and review of medical records for demographic, clinical and pathology information. A total of 5,831 breast cancer samples, which underwent prospective genomic profiling by the MSK-IMPACT targeted sequencing panel^9,63,64^ from January 2014 to December 2021, were retrieved. After removing samples with a sequencing-estimated tumor purity <20% (see below), a total of 3,880 breast cancer samples from 3,117 patients were included. Demographics, pathologic and detailed clinical information was collected until date of data freeze (June 2022).

Histological subtypes, tumor stage at diagnosis, tumor grade of primary breast cancer and receptor status were determined as described in Razavi et al.^9^. Briefly, breast cancer histological subtypes were classified as either invasive ductal carcinoma, invasive lobular carcinoma or mixed/other histologic types. Tumor grade was defined based on the Nottingham combined histologic grade of the primary breast cancer. The primary tumors with total tumor score of 3–5 were classified as G1 (well differentiated); 6–7 as G2 (moderately differentiated) and 8–9 as G3 (poorly differentiated). Patients were classified into breast cancer subtypes based on ER and progesterone receptor IHC results and the HER2 IHC and/or fluorescence in situ hybridization results rendered at the time of diagnosis in accordance with the American Society of Clinical Oncology and College of American Pathology guidelines^65,66^.

Each patient in our cohort was assigned a singular receptor status. Recognizing potential intertumoral heterogeneity, we sought a unified definition as follows: (1) in cases where any metastatic biopsy was sequenced, receptor status was defined by treating clinician interpretation and assigned first-line treatment; (2) in cases where only a primary tumor is sequenced, receptor status was defined by receptor status of the sequenced primary. Employing these definitions, our cohort consisted of 2,133 patients (68.4%) with HR^+^/HER2^−^ tumors, 276 (8.9%) with HR^+^/HER2^+^ tumors, 149 (4.8%) with HR^−^/HER2^+^ tumors and 559 patients (17.9%) with TNBC.

### MSK-IMPACT targeted sequencing analysis

Breast cancer samples underwent tumor-normal sequencing by the United States Food and Drug Administration-authorized MSK-IMPACT assay, which targets between 341 (2014) and 505 (2021) cancer-related genes. Genomic data extracted from MSK-IMPACT included somatic SNVs, CNAs, SVs and additional genomic metrics (TMB, mutations per Mb) and FGA (the percentage of the genome affected by CNAs^63,64^). Somatic mutations were classified as pathogenic, likely pathogenic or predicted oncogenic as defined by OncoKB annotation^67^. We used FACETS^68^ to define the allele-specific gene amplifications and homozygous deletions, tumor purity and ploidy, as previously described^69^. WGD status was inferred from MSK-IMPACT sequencing data, as previously described^69^. Briefly, tumor samples were considered to have undergone WGD if the fraction of major allele greater than one was >50%.

### Validation of SigMA performance on MSK-IMPACT and cell line WGS data

Although SigMA was designed originally to identify tumors positive for HRD, it has also proven effective in accurately classifying other types of mutational signatures, excelling particularly with APOBEC3 signatures^25^. SBS2 and SBS13 exhibit distinct trinucleotide context spectra, differing strongly from flat signatures like SBS3 and SBS5, or other specific signatures such as POLE and SMOKING, even with a minimal number of mutations for analysis. We thus aimed to independently assess the ability of SigMA to detect APOBEC3 signatures in breast cancer samples across three distinct datasets: TCGA breast cancers (primary, WES), Bertucci et al.^20^ (metastatic, WES) and Nik-Zainal et al.^28^ (primary, WGS). To validate SigMA, we adopted the same simulation approach as its developers, which involves MSK-IMPACT panel simulation.

This method is based on the concept that a high-quality signature evaluation requires a substantial mutation count, achievable only through WES and WGS. We initially computed signatures using real WES and WGS data, considering these results as the ground truth. Subsequently, we simulated MSK-IMPACT samples by reducing the mutations in each WES and WGS sample to only those within genomic regions covered by the MSK-IMPACT panel (version impact 468). SigMA (v.1.0.0.0) was then applied to these simulations to determine exposure and the dominant signature. Finally, we compared these outcomes with the established ground truth to obtain performance metrics, focusing on the ability to predict APOBEC3-dominant samples. Only samples with greater or equal to 5 SNVs in the simulated panel were considered for performance metric analysis.

### WGS analysis

To validate findings on MSK-IMPACT and provide further information regarding the evolution of APOBEC3 mutagenesis over time, WGS was carried out on five pairs of selected primary and MBC patient samples by the MSK’s Integrated Genomics Operations using validated protocols^25,27^. Briefly, microdissected tumor and germline DNA were subjected to WGS on HiSeq2000 (Illumina). The median sequencing coverage depth of 102× (range, 88×–132×) for tumor and 44× (range, 36×–58×) for normal samples. In addition, genomic DNA was extracted from 29 cell lines using the E.Z.N.A Tissue DNA Extraction Systems (Omega Bio-Tek, cat. no. 101319-018) and subjected to WGS, with a median sequencing coverage depth of 63× (for T47D lines) or 33× (for BT-474 lines) (range, 28×–103×).

For samples and cell lines subjected to WGS, data were processed through a validated bioinformatics pipeline^25,27^. Initially, sequence reads were aligned to the human reference genome GRCh37 utilizing the Burrows-Wheeler Aligner (BWA, v.0.7.15)^70^. SNVs in both WGS and MSK-IMPACT analyses were identified using MuTect (v.1.0)^71^. Indels were detected by employing a suite of tools: Strelka (v.2.0.15)^72^, VarScan2 (v.2.3.7)^73^, Platypus (v.0.8.1)^74^, Lancet (v.1.0.0)^75^ and Scalpel (v.0.5.3)^76^. CNAs and LOH assessments were conducted using FACETS (v.0.5.6)^68^. Mutations in tumor suppressor genes deemed deleterious/loss-of-function, or those targeting a known mutational hotspot in oncogenes, were classified as pathogenic. Hotspot-targeting mutations were annotated with reference to cancerhotspots.org (ref. ^77^).

Structural variants were identified using Manta (v.0.29.6)^78^, SvABA (v.1.1.0)^79^ and Gridss (v.2.13.2)^80^ from WGS data. Structural variants identified by at least two of the three callers were retained and utilized for subsequent analyses. Setup and call procedures are described in detail in their respective code repositories: for Manta, https://github.com/ipstone/modules/blob/master/sv_callers/mantaTN.mk and for SvABA, https://github.com/ipstone/modules/blob/master/sv_callers/svabaTN.mk. These processed SV calls, along with additional genomic data (SNVs, indels, CNAs), were integrated to create circos plots via the signature.tools.lib R package^81^ (code repository https://github.com/Nik-Zainal-Group/signature.tools.lib, v.2.4.0) using Rcircos (v.1.2.2) package. For all patients and cell lines with more than one sample, all unique variants from any samples in a given patient or cell line were genotyped in all other samples from the same patient or cell line using Waltz (v.3.2.0) (https://github.com/mskcc/Waltz).

### Mutational signature analysis

We have applied various tools to compute mutational signatures across different types of data. For WES data, DeconstructSigs (v.1.8.0)^31^, MutationalPatterns (v.3.4.1)^30^ and SigProfiler (v.0.0.25)^29^ were employed. For WGS data, we opted for Signal^82^ and, for the MSK-IMPACT panel, SigMA was used. The DeconstructSigs method is designed to identify the most accurate linear combination of predefined signatures that reconstructs a tumor sample’s mutational profile. This method employs a multiple linear regression model. DeconstructSigs—an R package extension—leverages the Bioconductor library at CRAN (https://cran.r-project.org/). MutationalPatterns operates as a non-negative least squares optimization algorithm. The non-negative least squares problem is studied extensively, and MutationalPatterns utilizes an R-based active set method from the pracma package (v.2.3.8) for its ‘fit_to_signatures’ function, available at CRAN. We ran MutationalPatterns with two different settings: ‘regular’ and ‘strict’. The ‘strict’ method suffers less from overfitting but can suffer from more signature misattribution. SigProfiler attributes a set of known mutational signatures to an individual sample, determining the activity of each signature and the probability of each causing specific mutation types. It integrates SigProfilerMatrixGenerator (v.1.2.5) for its functionality. We also used SigProfilerSimulator (v.1.1.4) and SigProfilerClusters (v.1.0.11) to assess the clustered mutations. Signal is considered one of the best state-of-the-art tool for WGS, offering a comprehensive workflow for mutational signature analysis. We set the number of bootstraps to 100, sparsity threshold type was fixed, sparsity threshold was 5% and sparsity *P* value was 0.05. Kataegis was detected using the Signal web portal (https://signal.mutationalsignatures.com).

SigMA can process samples with at least five somatic SNVs, making it particularly suitable for MSK-IMPACT samples. It encompasses five steps: discovery of mutational signatures in WGS data using NMF; clustering to determine tumor subtypes; simulation of cancer gene panels and exomes; calculation of likelihood, cosine similarity, and signature exposure; and training of gradient boosting classifiers for a final score. SigMA score thresholds are established based on simulated data, considering tumor type and sequencing platform.

The dominant mutational signature in each MSK-IMPACT sample, that is, the primary mutational process occurring in a cancer genome, was assessed using SigMA. We translated mutational exposures into percentages for cross-sample comparisons. For all other tools used in WGS and WES data, the dominant signature was defined as the mutation process with the highest percentage of exposure. This included groupings as Clock (SBS1 + SBS5), APOBEC3 (SBS2 + SBS13), HRD (SBS3 + SBS8) and considering Other signatures independently (for example, SBS17, SBS18, and so on). The process with the maximum exposure was considered dominant. To determine whether a mutation was APOBEC3-context, we identified characteristic peaks from cosmic signatures related to SBS2 (C>T mutations in the contexts TCA, TCC, TCG, TCT) and SBS13 (C>G mutations in the contexts TCA, TCC, TCG, TCT; C>A mutations in the contexts TCA, TCC, TCG, TCT), as described previously^25^.

### Treatment outcome analysis

All patients included in the treatment outcome analyses were treated at MSKCC. The exact regimen, dates of start and stop therapy, as well as date of progression was annotated via expert review. Progression events were defined as (1) radiographic or clinical event prompting change in systemic therapy or recommendation for locally targeted radiation therapy, (2) documented clinician impression detailing progression, after which there was documented patient or MD preference to continue same therapy, as previously described^9^.

We determined the association between dominant mutational signature and PFS with disease progression on therapy with ET ± CDK4/6 inhibitors or patient death. ETs, either as monotherapy or as partner of CDK4/6 inhibitors were categorized as following: aromatase inhibitor versus selective ER degrader. The log rank test was used to compare the survival distributions among two or more groups. Both univariate and multivariate Cox proportional hazard models (stratified by ET partner, and treatment line where available) were applied. For patients with several lines of therapy from the same class of treatment, only the first treatment line from that class that was started after the MSK-IMPACT biopsy was included in the analysis.

### Permutation analysis for APOBEC3 enrichment

To assess the correlation between APOBEC3 enrichment and the occurrence of SNVs within a gene, we adapted a method previously described^83^ for our dataset. The aim was to detect potential correlations between mutations in specific genes and APOBEC3 enrichment. To mitigate the impact of high TMB in APOBEC3-dominant samples, high frequency of mutations in longer genes, or the presence of high frequency genetic alterations in breast cancer, we applied a permutation-based method aimed at standardizing the overall mutation count for each sample and gene. This process randomized the gene × sample binary mutation matrix while preserving the mutation counts for each gene and sample, following the approach described by Strona et al.^84^. In this matrix, rows represent samples and columns represent genes, with ‘1’ indicating a mutation and ‘0’ indicating a wild-type nonmutated gene. We conducted an APOBEC3 enrichment analysis across all genes using a Wilcoxon rank sum *P* value to compare APOBEC3 exposure in mutant versus wild-type samples for each gene. To correct for multiple testing, *P* values were adjusted for the false discovery rate (FDR) using the Benjamini–Hochberg procedure. For the original dataset and each of 10,000 permutations, we calculated the *P* value_observed and *P* value_Random, respectively (for a total of 10,000 *P* value_Random). The final *P* value was calculated as the number of permutation iterations where *P* value_Random ≤ *P* value_observed, divided by the total number of permutations (10,000), ensuring a fair assessment of gene-mutation frequencies across samples with high mutation burdens. For the permutation analysis, we utilized the R package EcoSimR (v.0.1.0). The permutation test was carried out under various conditions, separately for HR^+^/HER2^−^ and for TNBC in both primary and metastatic settings, using pathogenic and likely pathogenic SNPs by OncoKB annotation. For each data group, we filtered for genes mutated in at least 1% of the subset obtained and for samples with at least one mutation in one of these genes. This filtering was necessary to ensure the EcoSimR package performed correctly during the randomization process. To compute *P* values, odds ratio, FDRs and other statistics we used different functions from the Python packages scipy (v.1.10.0) and statsmodels (v.0.13.2), whereas for visualization we used matplotlib.

### Genomic analysis of patients with several samples collected over time

To investigate the evolution of mutational processes over time, we subset the initial cohort of 3,800 breast cancers from MSK-IMPACT identifying patients with HR^+^/HER2^−^ subtype and several tumor samples collected over time and various treatments (patients with two samples, *n* = 449; patients with three or more samples, *n* = 43). Tumor sample pairs with complete somatic mismatches on the levels of SNVs, indels and/or CNAs were excluded.

To assess whether a somatic mutation was truly acquired over time and exposure to therapy, somatic mutations identified in samples collected afterward were interrogated in the matched respective primary tumor/first metastatic biopsy in a matched tumor-informed manner (genotyping) using Waltz (https://github.com/mskcc/Waltz), which required at least two duplex consensus reads, comprising both strands of DNA, to call a somatic SNV at a site known to be altered in the matched tumor sample from a given patient, as described previously^85^.

### Immunohistochemical analysis

Immunohistochemical analyses were conducted on a Leica Bond III automated stainer platform. Formalin-fixed paraffin-embedded (FFPE; 4 μm thick) tissue sections were subjected to heat-based antigen retrieval for 30 min using a high pH buffer solution (Bond Epitope Retrieval Solution 2; Leica, cat. no. AR9640). Subsequently, they were incubated with the A3A-13 (LQR-2-13 (UMN-13)) or the A3B (5210-87-13) primary antibodies at a 1:2,500 and 1:200 dilution, respectively for 30 min. A polymer detection system (Bond Polymer Refine Detection; Leica, cat. no. DS9800) was used as secondary reagent. Extent (percentage of tumor cells) and intensity (weak, moderate, strong) of A3A and A3B expression was evaluated was evaluated by two pathologists (F.P. and J.S.R.-F.).

### Cell lines

The following cell lines were used: T47D (ATCC HTB-133), MCF7 (ATCC HTB-22), BT-474 WT and *A3A* KO, and MDA-MB-453 WT and *A3A* KO (a gift from J. Maciejowski) and HEK293T (ATCC CRL-3216, a gift from P. Chi). T47D cells were cultured in RPMI medium; MCF7, BT-474 and MDA-MB-453 were cultured in DMEM/F12 medium; HEK293T were cultured in DMEM medium. All media were supplemented with 10% fetal bovine serum (Corning, cat. no. 35-010-CV), 2 mM l-glutamine, 100 U ml^−1^ penicillin and 100 µg ml^−1^ streptomycin (Gemini Bio 400-109). Tetracycline-free fetal bovine serum (Takara Bio, cat. no. 631367) was used for experiments using the doxycycline-system. All cells were maintained in a humidified incubator with 5% CO_2_ at 37 °C. All cell lines were routinely tested and were negative for mycoplasma contamination.

### Cloning and lentiviral transduction

The A3A^WT^, A3A^E72Q^, A3B^WT^ and A3B^E255Q^ sequences (cDNA plasmids were a gift from J. Maciejowski) were cloned into pDONR221 (Invitrogen, cat. no. 12536017) using the Gateway BP Clonase II Enzyme mix (Invitrogen, cat. no. 11789020). Expression vectors were generated by cloning into pInducer20 (a gift from S. Elledge, Addgene plasmid 4401; ref. ^86^) using the Gateway LR Clonase II enzyme mix (Invitrogen, cat. no. 11791020). Lentiviral particles were prepared by transfecting 293T cells with expression clones along with the lentiviral envelope and packaging plasmids using X-tremeGENE HP DNA transfection reagent (Sigma-Aldrich, cat. no. 6366546001). The medium was refreshed after 24 h. After 48 h, the supernatant was collected and filtered through 0.45 µm filters. T47D and MCF7 cells were transduced with the lentiviral particles and stably expressing cells were generated after selection using G418 (InvivoGen, cat. no. ant-gn-1) for 2 weeks. Cells were treated with 0.1 µg ml^−1^ doxycycline (Sigma-Aldrich, cat. no. D9891) to induce overexpression.

### Generation of resistant cells

T47D dA3A^WT^-5 and dA3A^E72Q^-5 cells were treated with DMSO or 500 nM abemaciclib for 14 days, after which the drug concentration was increased to 1 µM. Surviving dA3A^WT^-5 cells were tested for resistance after 2 months of continuous culture in 1 µM abemaciclib. dA3A^E72Q^-5 cells did not grow out even after 5 months under selection. Additionally, resistant cells were expanded after selection from the long-term growth assays.

### Antibodies and reagents

The following primary antibodies were obtained from Cell Signaling Technology and used for immunoblotting at a dilution of 1:1,000: anti-HA (C29F4), anti-p-Rb S780 (D59B7), anti-p-Rb S807/811 (D20B12), anti-Rb (4H1), anti-E2F1 (3742), anti-Cyclin E2 (4132S), anti-Cyclin A2 (BF683), anti-YAP1 (D8H1X), anti-β-tubulin (D3U1W) and anti-Vinculin (E1E9V). Anti-Vinculin (V9131) was obtained from Sigma-Aldrich and used at a dilution of 1:1,000. The secondary antibodies used were anti-Rabbit IgG, HRP-linked (1:3,000, Cell Signaling Technology, cat. no. 7074), anti-Rabbit IgG IRDye 680 RD (1:10,000, LI-COR Biosciences, cat no. 926-68071), and anti-Mouse IgG IRDye 800 RD (1:10,000, LI-COR Biosciences, cat. no. 926-32210). The following primary antibodies were obtained from R. Harris and used for IHC: anti-A3A-13 (1:2,500, cat no. LQR-2-13 (UMN-13)) and anti-A3A/B/G (1:200, cat. no. 5210-87-13). The following drugs were purchased from Selleck Chemicals: fulvestrant (S1191), abemaciclib (S5716), palbociclib (S1579), lapatinib (S2111), neratinib (S2150) and MK2206 (S1078), and dissolved in DMSO. Trastuzumab deruxtecan (Daiichi Sankyo, cat. no. DS-8201a) was dissolved in saline.

### Immunoblotting

Cells were lysed on ice using RIPA lysis buffer (Thermo Scientific, cat. no. 89901) supplemented with protease and phosphatase inhibitor (Thermo Scientific, cat. no. 78444). Lysates were cleared using centrifugation and total protein concentration was measured with BCA protein assay (Thermo Scientific, cat. no. 23225). Total protein 30–50 µg was separated on 4–12% Bis-Tris protein gels (Invitrogen NuPAGE) and transferred onto polyvinylidene fluoride or nitrocellulose membranes. Blots were blocked with Intercept (TBS) Blocking Buffer (LI-COR Biosciences, cat. no. 927-60001) or 5% milk, and incubated with primary antibodies overnight at 4 °C. After incubation with secondary antibodies, the blots were scanned by Odyssey CLx Imaging System (LI-COR Biosciences) or developed using the Western Lightning Plus-ECL (PerkinElmer, cat. no. NEL104001EA) and processed using Fiji v.2.0.0.

### DNA deaminase assay

In vitro deamination assay was performed as described previously^87^. Briefly, 50 µg total cell lysates were incubated with RNase A (1.75 U), ssDNA substrate (4 pmol), UDG buffer and UDG (1.25 U) in HED buffer (25 mM Hepes pH 7.8, 5 mM EDTA, 10% glycerol, 1 mM dithiothreitol freshly supplemented with protease and phosphatase inhibitor) for 2 h at 37 °C; 100 mM NaOH was then added and heated at 95 °C for 10 min to cleave the DNA at abasic sites. The sample was then heated with Novex Hi-Density TBE sample buffer (Invitrogen, cat. no. LC6678) at 95 °C for 5 min, cooled down on ice and run on a 15% TBE-urea PAGE gel. Separated DNA fragments were imaged on an ImageQuant 800 (Amersham). The ssDNA oligo substrates were 5′-(6-FAM)-GCAAGCTGTTCAGCTTGCTGA for A3A^88^ and 5′-ATTATTATTATTCAAATGGAT-TTATTTATTTATTTATTTATTT-fluorescein for A3B.

### In vitro growth assays

For cell viability measurement, 500–2,000 cells per well were plated in triplicates in 96-well plates in complete medium with 10% FBS or tetracycline-approved serum for doxycycline-inducible models. After overnight incubation for attachment, cells were treated with drugs (day 0). Medium was replaced once every week and doxycycline was topped up twice every week for overexpression cells. Viability was measured using the redox-sensitive dye Resazurin (R&D Systems, cat. no. AR002; 25 µl per well incubated at 37 °C for 4 h and read at 544 nm excitation and 590 nm emission using SpectraMax M5 (Molecular Devices)) or brightfield imaging of attached cells using Incucyte S3 (Sartorius). IC_50_ values were calculated by nonlinear regression of inhibitor concentrations with response in GraphPad Prism v.9.4.1. For colony forming assays, 2 × 10^5^ MDA-MB-453 cells were plated in triplicates in six-well plates. The cells were treated with drugs after 24 h, and their media replaced with fresh drugs twice every week. The cells were fixed with 100% methanol when confluent, washed with water, stained with 0.5% crystal violet (Sigma-Aldrich, cat. no. C0775) in 25% methanol, washed with water, dried and scanned using the AxioObserver 7 inverted microscope (Zeiss).

### Statistical analyses

Statistical analyses were conducted using R (v.3.1.2, v.4.1.0 and v.4.3.2) and GraphPad Prism v.9.4.1. Summary statistics were used to describe the study population. Fisher’s exact test or Pearson’s chi-squared test were used to compare categorical variables, whenever appropriate. Mann–Whitney *U* or Wilcoxon rank sum, or two-way analysis of variance (ANOVA) tests were used to compare continuous variables. A log rank test was used to compare the survival distributions between groups. Comparisons of frequencies of genes altered by somatic SNVs and CNAs as well as for site-specific gene alterations were performed using the Fisher’s exact test and logistic regression. Multiple testing correction using the Benjamini–Hochberg method was applied to control for the FDR whenever appropriate. Pearson’s coefficient *R* was computed using the python package scipy.stats (v.1.10.0). All *P* values were two-tailed, and 95% confidence intervals (CIs) were adopted for all analyses.

### Reporting summary

Further information on research design is available in the Nature Portfolio Reporting Summary linked to this article.

## Online content

Any methods, additional references, Nature Portfolio reporting summaries, source data, extended data, supplementary information, acknowledgements, peer review information; details of author contributions and competing interests; and statements of data and code availability are available at 10.1038/s41588-025-02187-1.

## Supplementary information

Reporting Summary

Supplementary Tables Supplementary Tables 1–7.

## Source data

Source Data Fig. 1 Uncropped and unprocessed blots and gels for Fig. 4k and Extended Data Figs. 3b,c and 7c.

## Data Availability

The MSK-IMPACT sequencing dataset is available through the cBioPortal for Cancer Genomics at http://www.cbioportal.org/study/summary?id=breast_msk_2025. WGS data from patient samples are available from the European Genome-Phenome Archive (EGA) (EGAD50000001275) under controlled access to protect patient confidentiality and adhere to ethical and legal standards for managing sensitive human genomic and phenotypic data. Qualified researchers may apply for data access through the EGA data access committee (DAC) EGAC50000000554, which will review requests to ensure they comply with participant consent and data use limitations. Sequencing data for cell line experiments is available on the Sequence Read Archive (SRA) under accession number PRJNA1231511. Data for breast cancers from TCGA were downloaded as the harmonized MC3 public MAF from https://gdc.cancer.gov/about-data/publications/mc3-2017, from Nik-Zainal et al.^28^ were download from the ICGC data portal (https://dcc.icgc.org; the portal was retired in June 2024, but the data remain accessible with controlled access requiring DAC approval (https://docs.icgc-argo.org/docs/data-access/icgc-25k-data) and raw data available from the EGA archive under ID EGAS00001001178) and data from Bertucci et al.^20^ (available from EGA under ID EGAS00001003290) were provided by F. André. Source data including comparison of signature assessment from different signature calling tools, quantification of mutational signatures and clustered mutations from WGS of cell lines and patient samples, and permutation tests for gene enrichment analyses are provided as supplementary tables. Source data are provided with this paper.

## Code availability

This study did not generate any custom code and all data were processed and analyzed using existing software whose details may be found in Methods.

## References

[1] Will, M., Liang, J., Metcalfe, C. & Chandarlapaty, S. Therapeutic resistance to anti-oestrogen therapy in breast cancer. *Nat. Rev. Cancer* **23**, 673–685 (2023). 37500767 10.1038/s41568-023-00604-3 PMC10529099

[2] Toy, W. et al. ESR1 ligand-binding domain mutations in hormone-resistant breast cancer. *Nat. Genet.* **45**, 1439–1445 (2013). 24185512 10.1038/ng.2822 PMC3903423

[3] Robinson, D. R. et al. Activating ESR1 mutations in hormone-resistant metastatic breast cancer. *Nat. Genet.* **45**, 1446–1451 (2013). 24185510 10.1038/ng.2823 PMC4009946

[4] Pearson, A. et al. Inactivating NF1 mutations are enriched in advanced breast cancer and contribute to endocrine therapy resistance. *Clin. Cancer Res.* **26**, 608–622 (2020). 31591187 10.1158/1078-0432.CCR-18-4044

[5] Sokol, E. S. et al. Loss of function of NF1 is a mechanism of acquired resistance to endocrine therapy in lobular breast cancer. *Ann. Oncol.* **30**, 115–123 (2019). 30423024 10.1093/annonc/mdy497 PMC6336006

[6] Nayar, U. et al. Acquired HER2 mutations in ER(+) metastatic breast cancer confer resistance to estrogen receptor-directed therapies. *Nat. Genet.* **51**, 207–216 (2019). 30531871 10.1038/s41588-018-0287-5

[7] Li, Z. et al. Loss of the FAT1 tumor suppressor promotes resistance to CDK4/6 inhibitors via the Hippo pathway. *Cancer Cell* **34**, 893–905.e8 (2018). 30537512 10.1016/j.ccell.2018.11.006 PMC6294301

[8] Condorelli, R. et al. Polyclonal RB1 mutations and acquired resistance to CDK 4/6 inhibitors in patients with metastatic breast cancer. *Ann. Oncol.* **29**, 640–645 (2018). 29236940 10.1093/annonc/mdx784

[9] Razavi, P. et al. The Genomic Landscape of Endocrine-Resistant Advanced Breast Cancers. *Cancer Cell* **34**, 427–438.e6 (2018). 30205045 10.1016/j.ccell.2018.08.008 PMC6327853

[10] Wander, S. A. et al. The genomic landscape of intrinsic and acquired resistance to cyclin-dependent kinase 4/6 inhibitors in patients with hormone receptor-positive metastatic breast cancer. *Cancer Discov.* **10**, 1174–1193 (2020). 32404308 10.1158/2159-8290.CD-19-1390 PMC8815415

[11] Razavi, P. et al. Alterations in PTEN and ESR1 promote clinical resistance to alpelisib plus aromatase inhibitors. *Nat. Cancer* **1**, 382–393 (2020). 32864625 10.1038/s43018-020-0047-1 PMC7450824

[12] Smith, A. E. et al. HER2^+^ breast cancers evade anti-HER2 therapy via a switch in driver pathway. *Nat. Commun.* **12**, 6667 (2021). 34795269 10.1038/s41467-021-27093-y PMC8602441

[13] Alexandrov, L. B. et al. The repertoire of mutational signatures in human cancer. *Nature* **578**, 94–101 (2020). 32025018 10.1038/s41586-020-1943-3 PMC7054213

[14] Pleasance, E. et al. Pan-cancer analysis of advanced patient tumors reveals interactions between therapy and genomic landscapes. *Nat. Cancer* **1**, 452–468 (2020). 35121966 10.1038/s43018-020-0050-6

[15] Koh, G., Degasperi, A., Zou, X., Momen, S. & Nik-Zainal, S. Mutational signatures: emerging concepts, caveats and clinical applications. *Nat. Rev. Cancer* **21**, 619–637 (2021). 34316057 10.1038/s41568-021-00377-7

[16] Harris, R. S. & Dudley, J. P. APOBECs and virus restriction. *Virology* **479-480**, 131–145 (2015). 25818029 10.1016/j.virol.2015.03.012 PMC4424171

[17] Sieuwerts, A. M. et al. Elevated APOBEC3B correlates with poor outcomes for estrogen-receptor-positive breast cancers. *Horm. Cancer* **5**, 405–413 (2014). 25123150 10.1007/s12672-014-0196-8 PMC4228172

[18] Law, E. K. et al. The DNA cytosine deaminase APOBEC3B promotes tamoxifen resistance in ER-positive breast cancer. *Sci. Adv.* **2**, e1601737 (2016). 27730215 10.1126/sciadv.1601737 PMC5055383

[19] Periyasamy, M. et al. APOBEC3B-mediated cytidine deamination is required for estrogen receptor action in breast cancer. *Cell Rep.* **13**, 108–121 (2015). 26411678 10.1016/j.celrep.2015.08.066 PMC4597099

[20] Bertucci, F. et al. Genomic characterization of metastatic breast cancers. *Nature* **569**, 560–564 (2019). 31118521 10.1038/s41586-019-1056-z

[21] Angus, L. et al. The genomic landscape of metastatic breast cancer highlights changes in mutation and signature frequencies. *Nat. Genet.* **51**, 1450–1458 (2019). 31570896 10.1038/s41588-019-0507-7 PMC6858873

[22] O’Leary, B. et al. The genetic landscape and clonal evolution of breast cancer resistance to Palbociclib plus Fulvestrant in the PALOMA-3 trial. *Cancer Discov.* **8**, 1390–1403 (2018). 30206110 10.1158/2159-8290.CD-18-0264 PMC6368247

[23] Cheng, D. T. et al. Memorial Sloan Kettering-Integrated Mutation Profiling of Actionable Cancer Targets (MSK-IMPACT): a hybridization capture-based next-generation sequencing clinical assay for solid tumor molecular oncology. *J. Mol. Diagn.* **17**, 251–264 (2015). 25801821 10.1016/j.jmoldx.2014.12.006 PMC5808190

[24] Gulhan, D. C., Lee, J. J., Melloni, G. E. M., Cortes-Ciriano, I. & Park, P. J. Detecting the mutational signature of homologous recombination deficiency in clinical samples. *Nat. Genet.* **51**, 912–919 (2019). 30988514 10.1038/s41588-019-0390-2

[25] Selenica, P. et al. APOBEC mutagenesis, kataegis, chromothripsis in EGFR-mutant osimertinib-resistant lung adenocarcinomas. *Ann. Oncol.* **33**, 1284–1295 (2022). 36089134 10.1016/j.annonc.2022.09.151 PMC10360454

[26] Batalini, F. et al. Mutational signature 3 detected from clinical panel sequencing is associated with responses to olaparib in breast and ovarian cancers. *Clin. Cancer Res.* **28**, 4714–4723 (2022). 36048535 10.1158/1078-0432.CCR-22-0749 PMC9623231

[27] Mandelker, D. et al. Genomic profiling reveals germline predisposition and homologous recombination deficiency in pancreatic acinar cell carcinoma. *J. Clin. Oncol.* **41**, 5151–5162 (2023). 37607324 10.1200/JCO.23.00561 PMC10667000

[28] Nik-Zainal, S. et al. Landscape of somatic mutations in 560 breast cancer whole-genome sequences. *Nature* **534**, 47–54 (2016). 27135926 10.1038/nature17676 PMC4910866

[29] Islam, S. M. A. et al. Uncovering novel mutational signatures by de novo extraction with SigProfilerExtractor. *Cell Genom.* **2**, 100179 (2022). 36388765 10.1016/j.xgen.2022.100179 PMC9646490

[30] Blokzijl, F., Janssen, R., van Boxtel, R. & Cuppen, E. MutationalPatterns: comprehensive genome-wide analysis of mutational processes. *Genome Med.* **10**, 33 (2018). 29695279 10.1186/s13073-018-0539-0 PMC5922316

[31] Rosenthal, R., McGranahan, N., Herrero, J., Taylor, B. S. & Swanton, C. DeconstructSigs: delineating mutational processes in single tumors distinguishes DNA repair deficiencies and patterns of carcinoma evolution. *Genome Biol.* **17**, 31 (2016). 26899170 10.1186/s13059-016-0893-4 PMC4762164

[32] Marcus, L. et al. FDA approval summary: pembrolizumab for the treatment of tumor mutational burden-high solid tumors. *Clin. Cancer Res.* **27**, 4685–4689 (2021). 34083238 10.1158/1078-0432.CCR-21-0327 PMC8416776

[33] Pareja, F. et al. The genomic landscape of metastatic histologic special types of invasive breast cancer. *npj Breast Cancer* **6**, 53 (2020). 33083532 10.1038/s41523-020-00195-4 PMC7560857

[34] Law, E. K. et al. APOBEC3A catalyzes mutation and drives carcinogenesis in vivo. *J. Exp. Med.* **217**, e20200261 (2020). 32870257 10.1084/jem.20200261 PMC7953736

[35] Cortez, L. M. et al. APOBEC3A is a prominent cytidine deaminase in breast cancer. *PLoS Genet.* **15**, e1008545 (2019). 31841499 10.1371/journal.pgen.1008545 PMC6936861

[36] Petljak, M. et al. Mechanisms of APOBEC3 mutagenesis in human cancer cells. *Nature* **607**, 799–807 (2022). 35859169 10.1038/s41586-022-04972-y PMC9329121

[37] Durfee, C. et al. Human APOBEC3B promotes tumor development in vivo including signature mutations and metastases. *Cell Rep. Med.* **4**, 101211 (2023). 37797615 10.1016/j.xcrm.2023.101211 PMC10591044

[38] Carpenter, M. A. et al. Mutational impact of APOBEC3A and APOBEC3B in a human cell line and comparisons to breast cancer. *PLoS Genet.* **19**, e1011043 (2023). 38033156 10.1371/journal.pgen.1011043 PMC10715669

[39] Naumann, J. A. et al. DNA deamination is required for human APOBEC3A-Driven hepatocellular carcinoma in vivo. *Int. J. Mol. Sci.* **24**, 9305 (2023). 37298259 10.3390/ijms24119305 PMC10253583

[40] Brown, W. L. et al. A rabbit monoclonal antibody against the antiviral and cancer genomic DNA mutating enzyme APOBEC3B. *Antibodies (Basel)* **8**, 47 (2019). 31544853 10.3390/antib8030047 PMC6783943

[41] Burns, M. B. et al. APOBEC3B is an enzymatic source of mutation in breast cancer. *Nature* **494**, 366–370 (2013). 23389445 10.1038/nature11881 PMC3907282

[42] Roberts, S. A. et al. An APOBEC cytidine deaminase mutagenesis pattern is widespread in human cancers. *Nat. Genet.* **45**, 970–976 (2013). 23852170 10.1038/ng.2702 PMC3789062

[43] Hirabayashi, S. et al. APOBEC3B is preferentially expressed at the G2/M phase of cell cycle. *Biochem. Biophys. Res. Commun.* **546**, 178–184 (2021). 33592502 10.1016/j.bbrc.2021.02.008

[44] Petljak, M. et al. Characterizing mutational signatures in human cancer cell lines reveals episodic APOBEC mutagenesis. *Cell* **176**, 1282–1294.e20 (2019). 30849372 10.1016/j.cell.2019.02.012 PMC6424819

[45] Roelofs, P. A. et al. Aberrant APOBEC3B expression in breast cancer is linked to proliferation and cell cycle phase. *Cells* **12**, 1185 (2023). 37190094 10.3390/cells12081185 PMC10136826

[46] Jarvis, M. C., Ebrahimi, D., Temiz, N. A. & Harris, R. S. Mutation signatures including APOBEC in cancer cell lines. *JNCI Cancer Spectr.* **2**, pky002 (2018). 29888758 10.1093/jncics/pky002 PMC5993214

[47] Taylor, B. J. et al. DNA deaminases induce break-associated mutation showers with implication of APOBEC3B and 3A in breast cancer kataegis. *eLife* **2**, e00534 (2013). 23599896 10.7554/eLife.00534 PMC3628087

[48] Mas-Ponte, D. & Supek, F. DNA mismatch repair promotes APOBEC3-mediated diffuse hypermutation in human cancers. *Nat. Genet.* **52**, 958–968 (2020). 32747826 10.1038/s41588-020-0674-6 PMC7610516

[49] Maciejowski, J., Li, Y., Bosco, N., Campbell, P. J. & de Lange, T. Chromothripsis and kataegis induced by telomere crisis. *Cell* **163**, 1641–1654 (2015). 26687355 10.1016/j.cell.2015.11.054 PMC4687025

[50] Xu, G. et al. ARID1A determines luminal identity and therapeutic response in estrogen-receptor-positive breast cancer. *Nat. Genet.* **52**, 198–207 (2020). 31932695 10.1038/s41588-019-0554-0 PMC7341683

[51] Davis, A. A. et al. Genomic complexity predicts resistance to endocrine therapy and CDK4/6 inhibition in hormone receptor-positive (HR^+^)/HER2-negative metastatic breast cancer. *Clin. Cancer Res.* **29**, 1719–1729 (2023). 36693175 10.1158/1078-0432.CCR-22-2177 PMC10150240

[52] Barroso-Sousa, R. et al. Prevalence and mutational determinants of high tumor mutation burden in breast cancer. *Ann. Oncol.* **31**, 387–394 (2020). 32067680 10.1016/j.annonc.2019.11.010

[53] Yang, C. et al. Acquired CDK6 amplification promotes breast cancer resistance to CDK4/6 inhibitors and loss of ER signaling and dependence. *Oncogene* **36**, 2255–2264 (2017). 27748766 10.1038/onc.2016.379 PMC5393973

[54] Li, Q. et al. INK4 tumor suppressor proteins mediate resistance to CDK4/6 kinase inhibitors. *Cancer Discov.* **12**, 356–371 (2022). 34544752 10.1158/2159-8290.CD-20-1726 PMC8831444

[55] Andre, F. et al. Alpelisib for PIK3CA-mutated, hormone receptor-positive advanced breast cancer. *N. Engl. J. Med.* **380**, 1929–1940 (2019). 31091374 10.1056/NEJMoa1813904

[56] Marabelle, A. et al. Efficacy of pembrolizumab in patients with noncolorectal high microsatellite instability/mismatch repair-deficient cancer: results from the phase II KEYNOTE-158 study. *J. Clin. Oncol.* **38**, 1–10 (2020). 31682550 10.1200/JCO.19.02105 PMC8184060

[57] Vasan, N. et al. Double PIK3CA mutations in cis increase oncogenicity and sensitivity to PI3Kalpha inhibitors. *Science* **366**, 714–723 (2019). 31699932 10.1126/science.aaw9032 PMC7173400

[58] Lee, J. Y. et al. Lobular carcinomas in situ display intralesion genetic heterogeneity and clonal evolution in the progression to invasive lobular carcinoma. *Clin. Cancer Res.* **25**, 674–686 (2019). 30185420 10.1158/1078-0432.CCR-18-1103 PMC6726436

[59] Isozaki, H. et al. Therapy-induced APOBEC3A drives evolution of persistent cancer cells. *Nature* **620**, 393–401 (2023). 37407818 10.1038/s41586-023-06303-1 PMC10804446

[60] Caswell, D. R. et al. The role of APOBEC3B in lung tumor evolution and targeted cancer therapy resistance. *Nat. Genet.* **56**, 60–73 (2024). 38049664 10.1038/s41588-023-01592-8 PMC10786726

[61] Varkaris, A. et al. Allosteric PI3Kalpha inhibition overcomes on-target resistance to orthosteric inhibitors mediated by secondary PIK3CA mutations. *Cancer Discov.* **14**, 227–239 (2024). 37916958 10.1158/2159-8290.CD-23-0704 PMC10850944

[62] Cardoso, F. et al. KEYNOTE-756: phase III study of neoadjuvant pembrolizumab (pembro) or placebo (pbo) plus chemotherapy (chemo), followed by adjuvant pembro or pbo plus endocrine therapy (ET) for early-stage high-risk ER^+^/HER2e breast cancer. *Ann. Oncol.* **34**, S1260–S1261 (2023).

[63] Zehir, A. et al. Mutational landscape of metastatic cancer revealed from prospective clinical sequencing of 10,000 patients. *Nat. Med.* **23**, 703–713 (2017). 28481359 10.1038/nm.4333 PMC5461196

[64] Nguyen, B. et al. Genomic characterization of metastatic patterns from prospective clinical sequencing of 25,000 patients. *Cell* **185**, 563–575.e11 (2022). 35120664 10.1016/j.cell.2022.01.003 PMC9147702

[65] Wolff, A. C. et al. Human epidermal growth factor receptor 2 testing in breast cancer: American Society of Clinical Oncology/College of American Pathologists clinical practice guideline focused update. *J. Clin. Oncol.* **36**, 2105–2122 (2018). 29846122 10.1200/JCO.2018.77.8738

[66] Allison, K. H. et al. Estrogen and progesterone receptor testing in breast cancer: ASCO/CAP guideline update. *J. Clin. Oncol.* **38**, 1346–1366 (2020). 31928404 10.1200/JCO.19.02309

[67] Chakravarty, D. et al. OncoKB: a precision oncology knowledge base. *JCO Precis. Oncol.* **1**, 1–16 (2017). 10.1200/PO.17.00011 PMC5586540 28890946

[68] Shen, R. & Seshan, V. E. FACETS: allele-specific copy number and clonal heterogeneity analysis tool for high-throughput DNA sequencing. *Nucleic Acids Res.* **44**, e131 (2016). 27270079 10.1093/nar/gkw520 PMC5027494

[69] Weigelt, B. et al. Molecular characterization of endometrial carcinomas in black and white patients reveals disparate drivers with therapeutic implications. *Cancer Discov.* **13**, 2356–2369 (2023). 37651310 10.1158/2159-8290.CD-23-0546 PMC11149479

[70] Li, H. & Durbin, R. Fast and accurate long-read alignment with Burrows–Wheeler transform. *Bioinformatics* **26**, 589–595 (2010). 20080505 10.1093/bioinformatics/btp698 PMC2828108

[71] Cibulskis, K. et al. Sensitive detection of somatic point mutations in impure and heterogeneous cancer samples. *Nat. Biotechnol.* **31**, 213–219 (2013). 23396013 10.1038/nbt.2514 PMC3833702

[72] Saunders, C. T. et al. Strelka: accurate somatic small-variant calling from sequenced tumor-normal sample pairs. *Bioinformatics* **28**, 1811–1817 (2012). 22581179 10.1093/bioinformatics/bts271

[73] Koboldt, D. C. et al. VarScan 2: somatic mutation and copy number alteration discovery in cancer by exome sequencing. *Genome Res.* **22**, 568–576 (2012). 22300766 10.1101/gr.129684.111 PMC3290792

[74] Rimmer, A. et al. Integrating mapping-, assembly- and haplotype-based approaches for calling variants in clinical sequencing applications. *Nat. Genet.* **46**, 912–918 (2014). 25017105 10.1038/ng.3036 PMC4753679

[75] Narzisi, G. et al. Genome-wide somatic variant calling using localized colored de Bruijn graphs. *Commun. Biol.* **1**, 20 (2018). 30271907 10.1038/s42003-018-0023-9 PMC6123722

[76] Narzisi, G. et al. Accurate de novo and transmitted indel detection in exome-capture data using microassembly. *Nat. Methods* **11**, 1033–1036 (2014). 25128977 10.1038/nmeth.3069 PMC4180789

[77] Chang, M. T. et al. Identifying recurrent mutations in cancer reveals widespread lineage diversity and mutational specificity. *Nat. Biotechnol.* **34**, 155–163 (2016). 26619011 10.1038/nbt.3391 PMC4744099

[78] Chen, X. et al. Manta: rapid detection of structural variants and indels for germline and cancer sequencing applications. *Bioinformatics* **32**, 1220–1222 (2016). 26647377 10.1093/bioinformatics/btv710

[79] Wala, J. A. et al. SvABA: genome-wide detection of structural variants and indels by local assembly. *Genome Res.* **28**, 581–591 (2018). 29535149 10.1101/gr.221028.117 PMC5880247

[80] Cameron, D. L. et al. GRIDSS: sensitive and specific genomic rearrangement detection using positional de Bruijn graph assembly. *Genome Res.* **27**, 2050–2060 (2017). 29097403 10.1101/gr.222109.117 PMC5741059

[81] Quinlan, A. R. & Hall, I. M. BEDTools: a flexible suite of utilities for comparing genomic features. *Bioinformatics* **26**, 841–842 (2010). 20110278 10.1093/bioinformatics/btq033 PMC2832824

[82] Degasperi, A. et al. A practical framework and online tool for mutational signature analyses show inter-tissue variation and driver dependencies. *Nat. Cancer* **1**, 249–263 (2020). 32118208 10.1038/s43018-020-0027-5 PMC7048622

[83] Kim, J. et al. Somatic ERCC2 mutations are associated with a distinct genomic signature in urothelial tumors. *Nat. Genet.* **48**, 600–606 (2016). 27111033 10.1038/ng.3557 PMC4936490

[84] Strona, G., Nappo, D., Boccacci, F., Fattorini, S. & San-Miguel-Ayanz, J. A fast and unbiased procedure to randomize ecological binary matrices with fixed row and column totals. *Nat. Commun.* **5**, 4114 (2014). 24916345 10.1038/ncomms5114

[85] Ashley, C. W. et al. High-sensitivity mutation analysis of cell-free DNA for disease monitoring in endometrial cancer. *Clin. Cancer Res.* **29**, 410–421 (2023). 36007103 10.1158/1078-0432.CCR-22-1134 PMC9852004

[86] Meerbrey, K. L. et al. The pINDUCER lentiviral toolkit for inducible RNA interference in vitro and in vivo. *Proc. Natl Acad. Sci. USA* **108**, 3665–3670 (2011). 21307310 10.1073/pnas.1019736108 PMC3048138

[87] Carpenter, M. A. et al. Methylcytosine and normal cytosine deamination by the foreign DNA restriction enzyme APOBEC3A. *J. Biol. Chem.* **287**, 34801–34808 (2012). 22896697 10.1074/jbc.M112.385161 PMC3464582

[88] Langenbucher, A. et al. An extended APOBEC3A mutation signature in cancer. *Nat. Commun.* **12**, 1602 (2021). 33707442 10.1038/s41467-021-21891-0 PMC7952602

