# Publisher Correction: APOBEC3 mutagenesis drives therapy resistance in breast cancer

**DOI:** 10.1038/s41588-025-02471-0

**Published:** 2025-12-12

**Authors:** Avantika Gupta, Andrea Gazzo, Pier Selenica, Anton Safonov, Fresia Pareja, Edaise M. da Silva, David N. Brown, Hong Shao, Yingjie Zhu, Juber Patel, Juan Blanco-Heredia, Bojana Stefanovska, Michael A. Carpenter, Yanjun Chen, Isabella Vegas, Xin Pei, Denise Frosina, Achim A. Jungbluth, Marc Ladanyi, Giuseppe Curigliano, Britta Weigelt, Nadeem Riaz, Simon N. Powell, Pedram Razavi, Reuben S. Harris, Jorge S. Reis-Filho, Antonio Marra, Sarat Chandarlapaty

**Affiliations:** 1 Human Oncology and Pathogenesis Program, Memorial Sloan Kettering Cancer Center, New York, NY USA; 2 Department of Pathology and Laboratory Medicine, Memorial Sloan Kettering Cancer Center, New York, NY USA; 3 Department of Medicine, Memorial Sloan Kettering Cancer Center, New York, NY USA; 4 Department of Biochemistry and Structural Biology, University of Texas Health San Antonio, San Antonio, TX USA; 5 Howard Hughes Medical Institute, University of Texas Health San Antonio, San Antonio, TX USA; 6 Department of Radiation Oncology, Memorial Sloan Kettering Cancer Center, New York, NY USA; 7 Department of Oncology and Haemato-Oncology, University of Milano, Milan, Italy; 8 Early Drug Development for Innovative Therapies, European Institute of Oncology IRCSS, Milan, Italy; 9 Weill-Cornell Medical College, New York, NY USA

**Keywords:** Breast cancer, Translational research

Correction to: *Nature Genetics* 10.1038/s41588-025-02187-1, published online 16 May 2025.

In the version of the article initially published, in Fig. 3b, the median PFS for HRD was “14.9 (3.9–6.8)” but should have been “4.9 (3.9–6.8)” and has now been corrected in the HTML and PDF versions of the article.

