# Supplementary material for: APOBEC3 mutagenesis drives therapy resistance in breast cancer
Source: Nat Genet. 2025 May 16;57(6):1452–62. doi: 10.1038/s41588-025-02187-1 (PMC12165862; doi:10.1038/s41588-025-02187-1)
Supplement: Supplementary file 1 — Reporting Summary [file 41588_2025_2187_MOESM1_ESM.pdf]

nature portfolio

Corresponding author(s): Antonio Marra, Sarat Chandarlapaty

Last updated by author(s): Mar 18, 2025

Reporting Summary

Nature Portfolio wishes to improve the reproducibility of the work that we publish. This form provides structure for consistency and transparency in reporting. For further information on Nature Portfolio policies, see our Editorial Policies and the Editorial Policy Checklist.

Statistics

For all statistical analyses, confirm that the following items are present in the figure legend, table legend, main text, or Methods section.

n/a	Confirmed	
☐	☒	The exact sample size (*n*) for each experimental group/condition, given as a discrete number and unit of measurement
☐	☒	A statement on whether measurements were taken from distinct samples or whether the same sample was measured repeatedly
☐	☒	The statistical test(s) used AND whether they are one- or two-sided<br>*Only common tests should be described solely by name; describe more complex techniques in the Methods section.*
☐	☒	A description of all covariates tested
☐	☒	A description of any assumptions or corrections, such as tests of normality and adjustment for multiple comparisons
☐	☒	A full description of the statistical parameters including central tendency (e.g. means) or other basic estimates (e.g. regression coefficient) AND variation (e.g. standard deviation) or associated estimates of uncertainty (e.g. confidence intervals)
☐	☒	For null hypothesis testing, the test statistic (e.g. *F*, *t*, *r*) with confidence intervals, effect sizes, degrees of freedom and *P* value noted<br>*Give P values as exact values whenever suitable.*
☒	☐	For Bayesian analysis, information on the choice of priors and Markov chain Monte Carlo settings
☒	☐	For hierarchical and complex designs, identification of the appropriate level for tests and full reporting of outcomes
☐	☒	Estimates of effect sizes (e.g. Cohen's *d*, Pearson's *r*), indicating how they were calculated

Our web collection on statistics for biologists contains articles on many of the points above.

Software and code

Policy information about availability of computer code

Data collection

A total of 3,880 breast cancer samples from 3,117 patients were included in the study. Tumor samples underwent prospective genomic profiling by the Memorial Sloan Kettering-Integrated Mutation Profiling of Actionable Cancer Targets (MSK-IMPACT) targeted sequencing panel from January 2014 and December 2021, were retrieved. Demographics, pathologic and detailed clinical information was collected until date of data freeze (June 2022). The exact regimen, dates of start and stop therapy, as well as date of progression was annotated via expert review.
WGS of cell lines and paired patient samples was carried by the MSKCC's Integrated Genomics Operations on HiSeq2000 (Illumina) using protocols described earlier (DOI https://doi.org/10.1016/j.annonc.2022.09.151). Immunohistochemical analyses were conducted on a Leica Bond III automated stainer platform (Leica, Deer Park, IL). Growth assay plates were read using SpectraMax M5 (Molecular Devices) or Incucyte S3 (Sartorius). Crystal violet plates were scanned using AxioObserver 7 (Zeiss). Immunoblots were imaged on iBright CL1000 (Invitrogen) or Odyssey CLX (LI-COR). DNA Deaminase assay gels were imaged using ImageQuant 800 (Amersham).

Data analysis

Genomic data extracted from MSK-IMPACT included somatic single nucleotide variants, copy number alterations (CNAs), structural variants, and additional genomic metrics as tumor mutational burden (TMB, Mut/Mb) and fraction of genome altered (FGA; i.e., the percentage of the genome affected by CNAs). Somatic mutations were classified as pathogenic, likely pathogenic or predicted oncogenic as defined by OncoKB annotation. We used FACETS to define the allele-specific gene amplifications and homozygous deletions, tumor purity, and ploidy. Whole genome doubling (WGD) status was inferred from MSK-IMPACT sequencing data. For samples and cell lines subjected to WGS, the data were processed through a validated bioinformatics pipeline. Initially, sequence reads were aligned to the human reference genome GRCh37 utilizing the Burrows-Wheeler Aligner (BWA, v0.7.15). SNVs in both WGS and MSK-IMPACT analyses were identified using MuTect (v1.0). Insertions and deletions (indels) were detected by employing a suite of tools: Strelka (v2.0.15), VarScan2 (v2.3.7), Platypus (v0.8.1), Lancet (v1.0.0), and Scalpel (v0.5.3). CNAs and LOH assessments were conducted using FACETS. Mutations in tumor suppressor genes deemed deleterious/loss-of-function, or those targeting a known mutational hotspot in oncogenes, were classified as pathogenic. Hotspot-targeting

mutations were annotated with reference to cancerhotspots.org. Structural variants were identified using Manta, SvABA and Gridss from WGS data. The processed structural variant calls, along with additional genomic data (SNVs, indels, CNAs), were integrated to create circos plots via the signature.tools.lib R package76 (code repository: https://github.com/Nik-Zainal-Group/signature.tools.lib). For all patients and cell lines with more than 1 sample, all unique variants from any samples in a given patient or cell line were genotyped in all other samples from the same patient or cell line using Waltz (https://github.com/mskcc/Waltz). Mutational signature analyses were conducted using DeconstructSigs, MutationalPatterns, and SigProfiler for WES data, Signal for WGS, and SigMA for MSK-IMPACT data. For the permutation analysis, we utilized the R package EcoSimR. To compute p values, odds ratio, false discovery rates and other statistics we used different functions from the Python packages scipy and statsmodels, while for visualization we used matplotlib.Extent (% of tumor cells) and intensity (weak, moderate, strong) of A3A and A3B IHC was evaluated was evaluated by two pathologists (FP and JSR-F). Immunoblots and DNA Deaminase assay gel images were analyzed using Fiji v2.0.0 or ImageStudio v5.2.5 (LI-COR). Data were plotted using GraphPad Prism v9.4.1 or R4.3.2. Statistical analyses were conducted using R (version 3.1.2) or GraphPad Prism v9.4.1. Summary statistics were used to describe the study population. Fisher's exact test was used to compare categorical variables. Mann–Whitney U or Wilcoxon rank sum, or two-way ANOVA tests were used to compare continuous variables. Comparisons of frequencies of genes altered by somatic genetic and CNAs were performed using the Fisher's exact test and logistic regression. Multiple testing correction using the Benjamini–Hochberg method was applied to control for the false discovery rate (FDR) whenever appropriate. Pearson's coefficient r was computed using the python package scipy.stats. All p values were two-tailed, and 95% confidence intervals were adopted for all analyses. Figures were assembled using Illustrator 2024 (Adobe).

All software used in this manuscript are available online at:
R 3.1.2, 4.1.0, 4.3.2
OncoKB (https://www.oncokb.org)
cancerhotspots (https://www.cancerhotspots.org/#/home)
Burrows-Wheeler Aligner (BWA, v0.7.15) (https://github.com/lh3/bwa)
MuTect (v1.0) (https://github.com/broadinstitute/mutect)
Strelka (v2.0.15) (https://github.com/Illumina/strelka)
VarScan2 (v2.3.7) (https://github.com/dkoboldt/varscan)
Platypus (v0.8.1) (https://github.com/AdamaJava/adamajava)
Lancet (v1.0.0) (https://github.com/nygenome/lancet)
Scalpel (v0.5.3) (https://github.com/KarchinLab/scalpel)
FACETS (v0.5.6) (https://github.com/mskcc/facets)
Manta (v0.29.6) (https://github.com/Illumina/manta)
SvABA (v1.1.0) (https://github.com/walaj/svaba)
Gridss (v2.13.2) (https://github.com/PapenfussLab/gridss)
Waltz (v3.2.0) (https://github.com/mskcc/Waltz)
Rcircos (v1.2.2) (https://cran.r-project.org/web/packages/RCircos/index.html)
DeconstructSigs (v1.8.0) (https://github.com/raerose01/deconstructSigs)
MutationalPatterns (v3.4.1) (https://github.com/UMCUGenetics/MutationalPatterns)
SigProfiler (v0.0.25) (https://github.com/AlexandrovLab/SigProfilerExtractor)
Signal (https://signal.mutationalsignatures.com)
SigMA (v1.0.0.0) (https://github.com/getzlab/sigma)
SigProfilerClusters (v1.0.11) (https://github.com/AlexandrovLab/SigProfilerClusters)
SigProfilerSimulator (v1.1.4) (https://github.com/AlexandrovLab/SigProfilerSimulator)
SigProfilerMatrixGenerator (v1.2.5) (https://github.com/AlexandrovLab/SigProfilerMatrixGenerator)
EcoSimR (v0.1.0) (https://cran.r-project.org/src/contrib/Archive/EcoSimR/)
signature.tools.lib (v2.4.0) (https://github.com/Nik-Zainal-Group/signature.tools.lib)
pracma (v2.3.8) (https://github.com/cran/pracma)
scipy (v1.10.0) (https://scipy.org)
scipy.stats (v1.10.0) (https://scipy.org)
statsmodels (v0.13.2) (https://www.statsmodels.org/stable/index.html)

For manuscripts utilizing custom algorithms or software that are central to the research but not yet described in published literature, software must be made available to editors and reviewers. We strongly encourage code deposition in a community repository (e.g. GitHub). See the Nature Portfolio guidelines for submitting code & software for further information.

Data

Policy information about availability of data

All manuscripts must include a data availability statement. This statement should provide the following information, where applicable:

- Accession codes, unique identifiers, or web links for publicly available datasets
- A description of any restrictions on data availability
- For clinical datasets or third party data, please ensure that the statement adheres to our policy

The MSK-IMPACT sequencing dataset is available through the cBioPortal for Cancer Genomics at http://www.cbioportal.org/study/summary?id=breast_msk_2025. WGS data from patient samples is available from the European Genome-Phenome Archive (EGA) (EGAD50000001275) with required access from data access committee (DAC) EGAC50000000554. Sequencing data for cell line experiments is available on the Sequence Read Archive (SRA) under accession number PRJNA1231511. Data for breast cancers from TCGA were downloaded as the harmonized MC3 public MAF from https://gdc.cancer.gov/about-data/publications/mc3-2017, from Nik-Zainal et al. (DOI https://doi.org/10.1038/nature17676) were download from the ICGC data portal (https://dcc.icgc.org; the portal was retired in June 2024, but the data remain accessible with controlled access requiring DAC approval (https://docs.icgc-argo.org/docs/data-access/icgc-25k-data) and raw data available from the EGA archive under ID EGAS00001001178), and data from Bertucci et al. (DOI https://doi.org/10.1038/s41586-019-1056-z) were provided by Dr. Fabrice André. Source data including comparison of signature assessment from different signature calling tools, quantification of mutational signatures and clustered mutations from WGS of cell lines and patient samples, and permutation tests for gene-enrichment analyses are provided as supplementary material.

Research involving human participants, their data, or biological material

Policy information about studies with human participants or human data. See also policy information about sex, gender (identity/presentation), and sexual orientation and race, ethnicity and racism.

Reporting on sex and gender	Male patients composed 0.9% (n = 29) of this breast cancer cohort (otherwise composed of n = 3,117 total patients, 3,088 females). Hence, we did not separately include sex as a covariate in clinical outcome or genomic enrichment analyses.
Reporting on race, ethnicity, or other socially relevant groupings	We did not report race or ethnicity characteristics in this version of the manuscript. This information is available at https://www.cbioportal.org/study/summary?id=breast_msk_2025.
Population characteristics	We did not report on age in this manuscript. Sex, race and ethnicity information is available at https://www.cbioportal.org/study/summary?id=breast_msk_2025.
Recruitment	The study cohort comprised 3,880 tumor samples from 3,117 patients who had a diagnosis of breast cancer. All patients underwent prospective clinical tumor and germline sequencing as part of their clinical care (January 2014 to December 2021).
Ethics oversight	The study was approved by the MSKCC Institutional Review Board (12-245).

Note that full information on the approval of the study protocol must also be provided in the manuscript.

Field-specific reporting

Please select the one below that is the best fit for your research. If you are not sure, read the appropriate sections before making your selection.

☒ Life sciences ☐ Behavioural & social sciences ☐ Ecological, evolutionary & environmental sciences

For a reference copy of the document with all sections, see nature.com/documents/nr-reporting-summary-flat.pdf

Life sciences study design

All studies must disclose on these points even when the disclosure is negative.

Sample size	The study cohort comprised 3,880 tumor samples from 3,117 patients who had a diagnosis of breast cancer. All patients underwent prospective clinical tumor and germline sequencing as part of their clinical care (January 2014 to December 2021). No statistical analysis was performed to pre-determine sample size.
Data exclusions	Samples with a sequencing-estimated tumor purity <20% were excluded from all analyses.<br>From the clinical CDK4/6i and ET analysis, samples were additionally excluded for the following reasons:<br>i) Receptor status TNBC or HER2+<br>ii) Cases receiving CDK4/6i for fewer than 2 weeks<br>iii) Oligometastatic tumors for which the only visible metastatic sites underwent local ablation with radiation therapy or surgery prior to initiation of CDK4/6i.
Replication	All experimental findings were confirmed by multiple biological replicates as indicated in the figure legends.
Randomization	For the analyses including clinical samples from patients, randomization was not performed. Covariates were controlled using multivariate logistic regressions. For experiments using cell lines, different replicates in the control or experimental groups originated from the same starting population for individual experiments.
Blinding	For the analyses including clinical samples from patients, blinding was not performed, as there were no subjective judgements involved. For experiments using cell lines, the experimental groups could not be blinded due to the periodic replenishment of media and drugs.

Reporting for specific materials, systems and methods

We require information from authors about some types of materials, experimental systems and methods used in many studies. Here, indicate whether each material, system or method listed is relevant to your study. If you are not sure if a list item applies to your research, read the appropriate section before selecting a response.

Materials & experimental systems

n/a	Involved in the study
☐	☒ Antibodies
☐	☒ Eukaryotic cell lines
☒	☐ Palaeontology and archaeology
☒	☐ Animals and other organisms
☐	☒ Clinical data
☒	☐ Dual use research of concern
☒	☐ Plants

Methods

n/a	Involved in the study
☒	☐ ChIP-seq
☒	☐ Flow cytometry
☒	☐ MRI-based neuroimaging

Antibodies

Antibodies used

The following primary antibodies were obtained from Cell Signaling Technology and used for immunoblotting at a dilution of 1:1000: anti-HA (C29F4), anti-p-Rb S780 (D59B7), anti-p-Rb S807/811 (D20B12), anti-Rb (4H1), anti-E2F1 (3742), anti-Cyclin E2 (4132S), anti-Cyclin A2 (BF683), anti-YAP1 (D8H1X), anti-β-tubulin (D3U1W), and anti-Vinculin (E1E9V). Anti-Vinculin (V9131) was obtained from Sigma-Aldrich and used at a dilution of 1:1000. The secondary antibodies used were anti-Rabbit IgG, HRP-linked (1:3000, Cell Signaling Technology 7074), anti-Rabbit IgG IRDye 680 RD (1:10000, LI-COR Biosciences 926-68071), and anti-Mouse IgG IRDye 800 RD (1:10000, LI-COR Biosciences 926-32210). The following primary antibodies were obtained from Dr. Reuben Harris, and used for IHC: anti-A3A-13 (1:2500, LQR-2-13 (UMN-13)), and anti-A3A/B/G (1:200, 5210-87-13).

Validation

The antibodies for Western Blotting were validated by the manufacturer (Cell Signaling Technology) using SimpleChIP Enzymatic Chromatin IP kits (rabbit anti-HA, mouse anti-Rb, rabbit anti-YAP1 and rabbit anti-E2F1) or by expected change in signal by serum-starvation (rabbit anti-p-Rb S780), phosphatase treatment (rabbit anti-p-Rb S807/811), proteasome inhibition (rabbit anti-Cyclin E2), cell cycle arrest (mouse anti-Cyclin A2) or varying expression in different cell lines (rabbit and mouse anti-Vinculin and mouse anti-β-tubulin). All bands in the immunoblots were of the reported sizes. Validation of the IHC antibodies has been described in Naumann et al. (DOI https://doi.org/10.3390/ijms24119305) and Brown et al. (DOI https://doi.org/10.3390/antib8030047).

Eukaryotic cell lines

Policy information about cell lines and Sex and Gender in Research

Cell line source(s)

The following cell lines were used: T47D (ATCC HTB-133), MCF7 (ATCC HTB-22), HEK293T (ATCC CRL-3216), BT-474 WT and A3A KO, MDA-MB-453 WT and A3A KO (Petljak et al. DOI https://doi.org/10.1038/s41586-022-04972-y).

Authentication

STR profile analysis was used for cell lines obtained from Petljak et al. (DOI https://doi.org/10.1038/s41586-022-04972-y). The other cell lines were not authenticated for this study.

Mycoplasma contamination

All cell lines were routinely tested negative for mycoplasma contamination.

Commonly misidentified lines
(See ICLAC register)

No commonly misidentified cell lines were used in this study.

Clinical data

Policy information about clinical studies

All manuscripts should comply with the ICMJE guidelines for publication of clinical research and a completed CONSORT checklist must be included with all submissions.

Clinical trial registration

NCT01775072.

Study protocol

The study protocol describing MSK-IMPACT is available at: https://clinicaltrials.gov/study/NCT01775072.

Data collection

A total of 3,880 breast cancer samples, which underwent prospective genomic profiling by the Memorial Sloan Kettering-Integrated Mutation Profiling of Actionable Cancer Targets (MSK-IMPACT) targeted sequencing panel from January 2014 and December 2021, were retrieved. The study was approved by the Memorial Sloan Kettering Cancer Center (MSKCC) Institutional Review Board (12-245) and all patients provided written informed consent for tumor sequencing and review of medical records for demographic, clinical and pathology information. Demographics, pathologic and detailed clinical information was collected until date of data freeze (June 2022).

Outcomes

This was a retrospective, observational study so primary and secondary outcomes were not pre-specified. We determined the association between APOBEC3 status and progression-free survival on therapy with ET +/- CDK4/6 inhibitors . Disease progression was defined as the date of the radiology study that prompted a change in systemic treatment, intervention with locally directed therapy (e.g. radiation therapy), or otherwise an annotation in the chart documenting progression of disease. We categorized CDK4/6 inhibitor regimens based on their endocrine therapy partner (aromatase inhibitor vs selective estrogen receptor degrader/SERD). We used both univariate and multivariate Cox proportional hazard models (stratified by endocrine therapy partner, and treatment line, where available). For patients with multiple lines of therapy from the same class of treatment, only the first treatment line from that class that was started after the MSK-IMPACT biopsy was included in the analysis.

Plants

Seed stocks	NA
Novel plant genotypes	NA
Authentication	NA
