# Supplementary material for: APOBEC3 mutagenesis drives therapy resistance in breast cancer
Source: Nat Genet. 2025 May 16;57(6):1452–62. doi: 10.1038/s41588-025-02187-1 (PMC12165862; doi:10.1038/s41588-025-02187-1)

Fig. 4k:

p-Rb S780

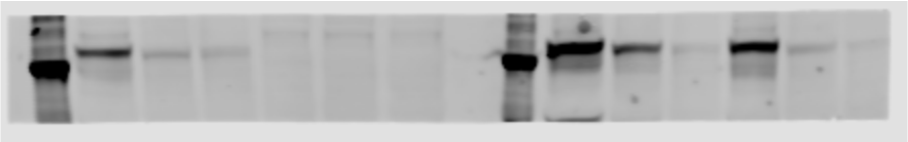

p-Rb S807/811

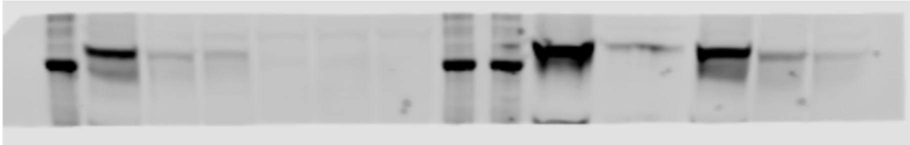

Rb

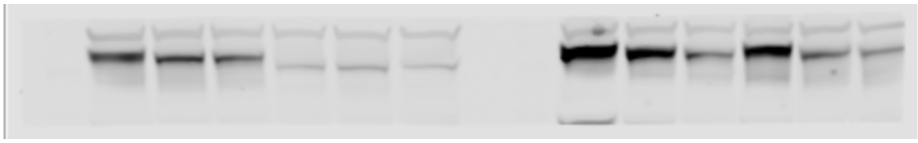

E2F1

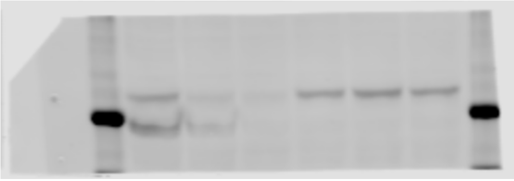

Cyclin E2

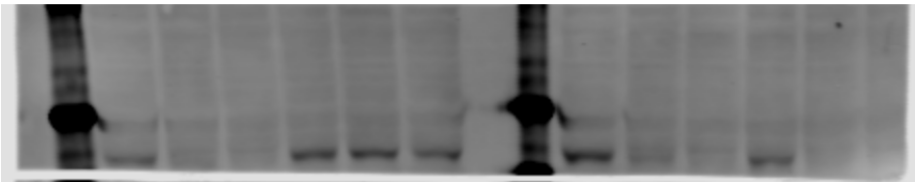

Cyclin A2

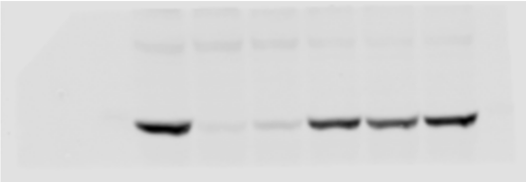

Vinculin

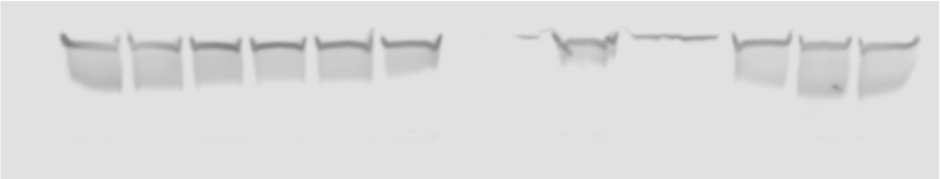

Extended Data Fig. 3b:

Immunoblots:

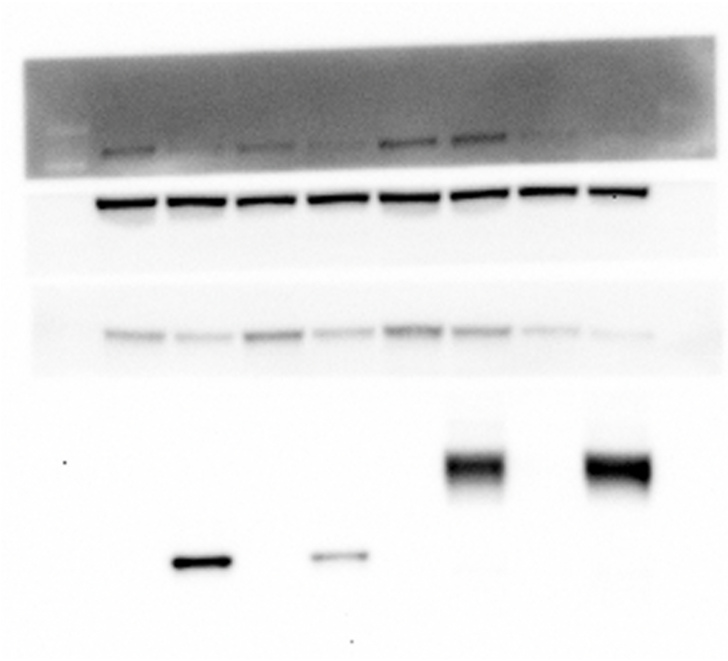

Vinculin

HA

Deaminase assay:

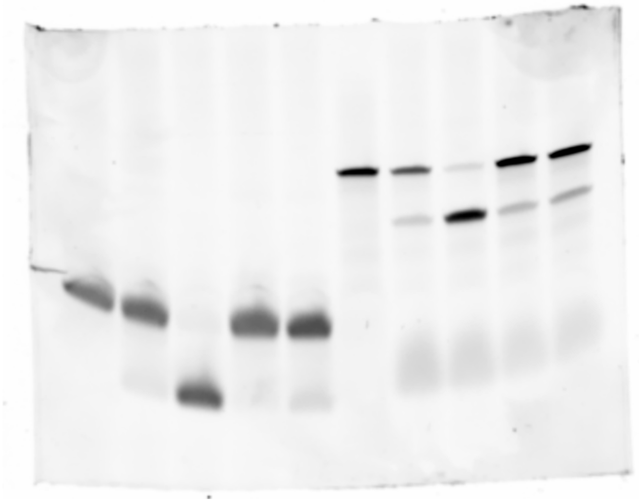

Extended Data Fig. 3c:

Immunoblots:

dA3A^{WT}:

Vinculin

HA

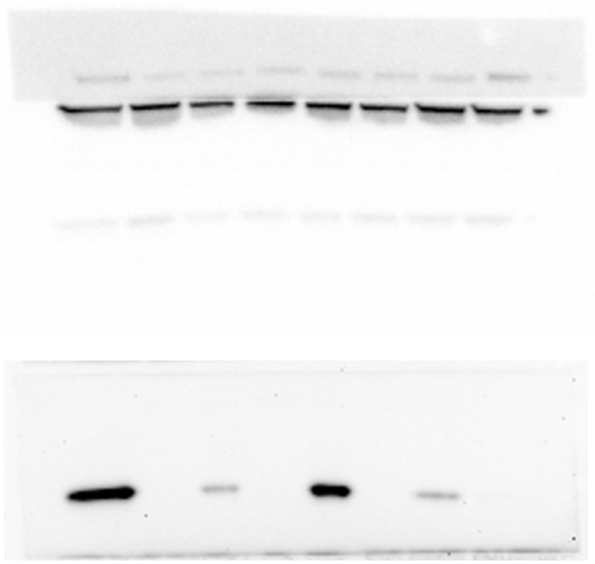

dA3A^{E72Q}:

Vinculin

HA

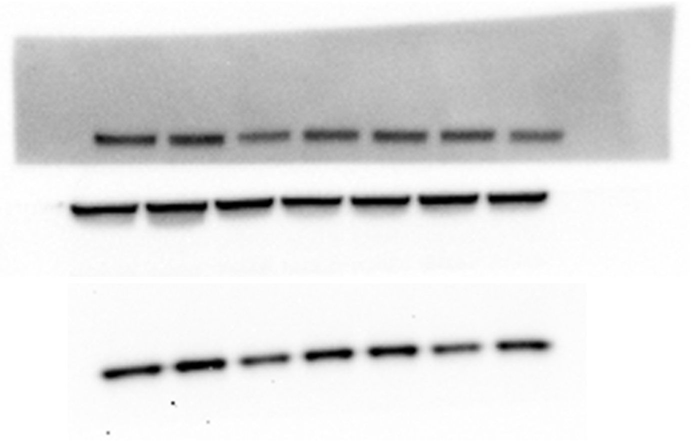

dA3B^{WT}:

Vinculin

HA

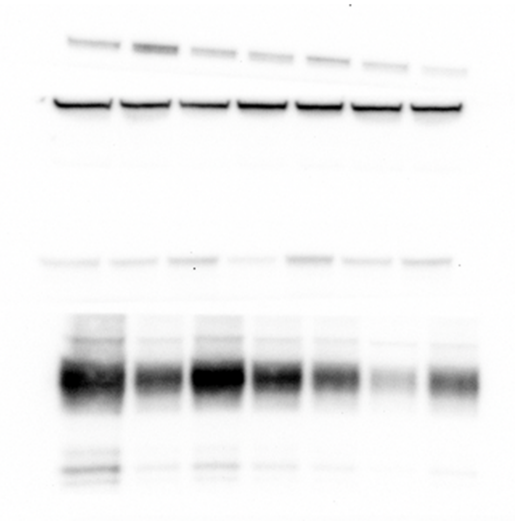

dA3B^{E255Q}:

Vinculin

HA

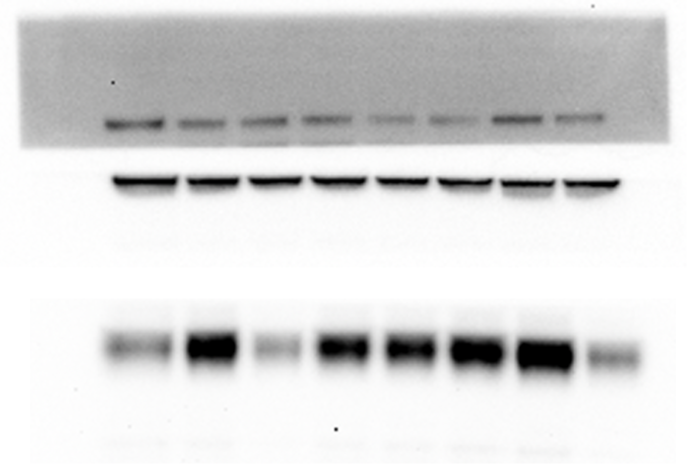

Deaminase assay:

A3A:

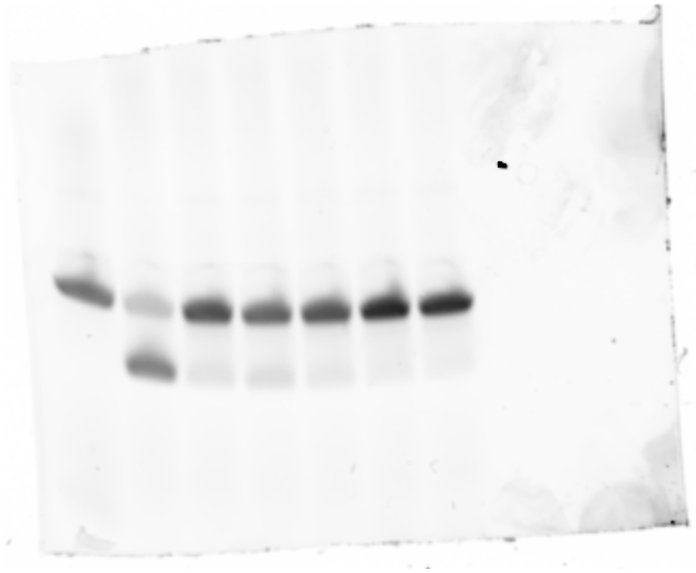

A3B:

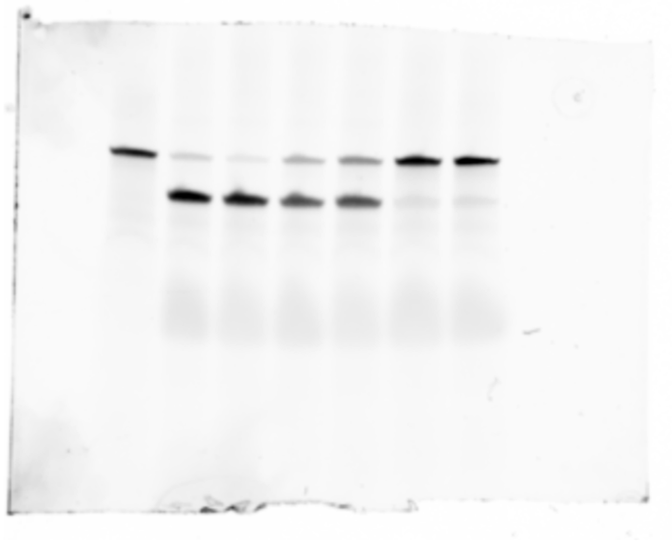

Extended Data Fig. 7c:

YAP1

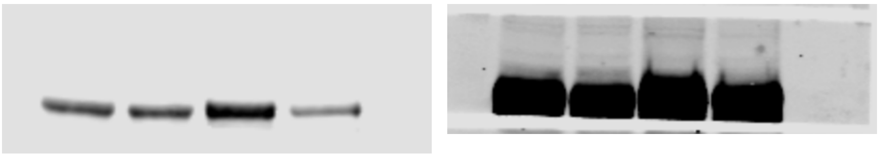

p-Rb S780

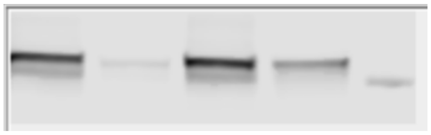

Rb

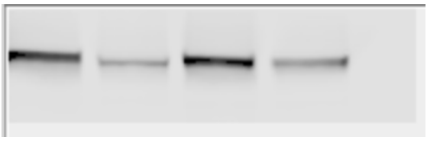

E2F1

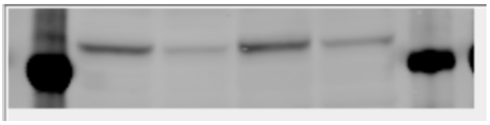

Cyclin A2

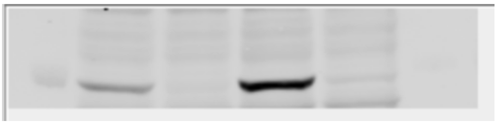

β-tubulin

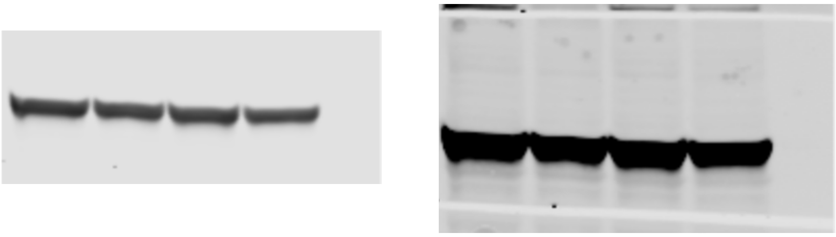

Supplement: Supplementary file 3 — Uncropped and unprocessed blots and gels for Fig. 4k and Extended Data Figs. 3b,c and 7c. [file 41588_2025_2187_MOESM3_ESM.pdf]
